# Pre-vaccination monocyte-to-lymphocyte ratio as a biomarker for the efficacy of malaria candidate vaccines: A subgroup analysis of pooled clinical trial data

**DOI:** 10.1371/journal.pone.0291244

**Published:** 2023-09-14

**Authors:** Jane Paula Nyandele, Ummi Abdul Kibondo, Fatuma Issa, Jean Pierre Van Geertruyden, George Warimwe, Said Jongo, Salim Abdulla, Ally Olotu

**Affiliations:** 1 Global Health Institute, University of Antwerp, Antwerp, Belgium; 2 Ifakara Health Institute, Bagamoyo Clinical Trial Unit, Bagamoyo, Tanzania; 3 KEMRI-Wellcome Trust Research Programme, Kilifi, Kenya; Para Federal University, BRAZIL

## Abstract

**Background:**

Pre-vaccination monocyte-to-lymphocyte ratio was previously suggested as a marker for malaria vaccine effectiveness. We investigated the potential of this cell ratio as a marker for malaria vaccine efficacy and effectiveness. Effectiveness was investigated by using clinical malaria endpoint, and efficacy was investigated by using surrogate endpoints of *Plasmodium falciparum* prepatent period, parasite density, and multiplication rates in a controlled human malaria infection trial (CHMI).

**Methods:**

We evaluated the correlation between monocyte-to-lymphocyte ratio and RTS,S vaccine effectiveness using Cox regression modeling with clinical malaria as the primary endpoint. Of the 1704 participants in the RTS,S field trial, data on monocyte-to-lymphocyte ratio was available for 842 participants, of whom our analyses were restricted. We further used Spearman Correlations and Cox regression modeling to evaluate the correlation between monocyte-to-lymphocyte ratio and Whole Sporozoite malaria vaccine efficacy using the surrogate endpoints. Of the 97 participants in the controlled human malaria infection vaccine trials, hematology and parasitology information were available for 82 participants, of whom our analyses were restricted.

**Results:**

The unadjusted efficacy of RTS,S malaria vaccine was 54% (95% CI: 37%-66%, p <0.001). No correlation was observed between monocyte-to-lymphocyte ratio and RTS,S vaccine efficacy (Hazard Rate (HR):0.90, 95%CI:0.45–1.80; p = 0.77). The unadjusted efficacy of Whole Sporozoite malaria vaccine in the appended dataset was 17.6% (95%CI:10%-28.5%, p<0.001). No association between monocyte-to-lymphocyte ratio and the Whole Sporozoite malaria vaccine was found against either the prepatent period (HR = 1.16; 95%CI:0.51–2.62, p = 0.72), parasite density (rho = 0.004, p = 0.97) or multiplication rates (rho = 0.031, p = 0.80).

**Conclusion:**

Monocyte-to-lymphocyte ratio alone may not be an adequate marker for malaria vaccine efficacy. Further investigations on immune correlates and underlying mechanisms of immune protection against malaria could provide a clearer explanation of the differences between those protected in comparison with those not protected against malaria by vaccination.

## Introduction

Between 2000 and 2019, malaria incidence rates have decreased by 27% globally and 40% in the WHO African region, and mortality rates fell by 57% globally and 63% in the African region [1]. The recent successes in control of malaria encouraged calls for elimination, but emerging parasite drug resistance and vector insecticide resistance are threatening the progress [2]. Malaria vaccines have a big potential to complement the existing strategies and accelerate progress towards malaria elimination. A number of pre-erythrocytic, erythrocytic and transmission-blocking malaria vaccines are developed, but all with limited efficacy [3]. While an ideal malaria vaccine should show at least 80% clinical efficacy, the most advanced vaccine, RTS,S, shows maximum efficacy of about 56% varying substantially between individuals and according to level of malaria exposure [4–6].

Assessing heterogeneity in natural malaria exposure and its impact is a key element in evaluating the effectiveness of antimalarial interventions, including malaria vaccines [7, 8]. A number of approaches are reported, including the use of weighted local parasite exposure indices [6, 9], entomological inoculation rates [10] and malaria-specific serological data [11, 12]. These approaches are often expensive, time-consuming, and lack precision.

Experimental malaria vaccine study followed by controlled human malaria infection (CHMI) holds potential for overcoming the challenges of heterogeneity to malaria exposure often encountered in field trials [13–15]. CHMI ensures a homogenous exposure in terms of malaria parasite load, strain and timing of infection. In addition, the clinical malaria endpoint is substituted with surrogate endpoints of parasite density, pre-patent period, and kinetic growth rate, which are quantitative and hence more objective [16].

In clinical trials, hematological and immunological markers bear potential to help stratify vaccine recipients that are most likely to be protected by a vaccine from those that are refractory to vaccine protection. Identifying predictors for low vaccine efficacy may lead to hypotheses and thus inform research strategies for improving vaccine performance [17].

Some cells of the immune system such as neutrophils, eosinophils, monocytes and lymphocytes are suggested markers for immune responses [18, 19]. Such cells play a dynamic role in balancing pro- and anti-inflammatory immune responses following an infection, which result in pathogen clearance while limiting damage to host tissues [19]. In response to an infection, monocytes release pro-inflammatory cytokines, which are then balanced by the adaptive responses of lymphocytes for an effective immune response that prevents excessive inflammation during the clearing of an infection [19–21]. The ratio of monocytes to lymphocytes reflects a balance of inflammatory immune activities and can thus be used as an index for an individuals’ immune status. A number of studies have reported a correlation between monocyte-to-lymphocyte ratio (ML ratio) and the extent of inflammatory diseases such as tuberculosis and cardiovascular disease [22–24], and has been identified as a diagnostic and prognostic marker for a wide range of diseases including osteoarthritis, colorectal and pancreatic cancer [25–27]. ML ratio has also been reported to correlate significantly with malaria parasitemia, as with the risk for clinical and severe malaria in children under five [18, 28]. Therefore, ML ratio has been suggested as a marker for the efficacy of RTS,S malaria vaccine, but further investigations are needed [17].

This study investigated whether peripheral blood ML ratio measured at study enrolment is a marker for the efficacy of candidate malaria vaccines in a secondary analysis from two reported vaccine trials. First, from reported pediatric RTS,S malaria vaccine trial with natural malaria parasites exposure and with clinical malaria as primary endpoint. Secondly, from reported attenuated *P*. *falciparum* sporozoite malaria vaccine studies followed by CHMI in adults with surrogate efficacy endpoints of *P*. *falciparum* parasite density, prepatent period and multiplication rates.

## Methods

### Study setting

This report is according to the Strengthening the Reporting of Observational Studies in Epidemiology (STROBE) guidelines (Additional file 1). The main aim was to relate pre-vaccination ML ratio to the efficacy of malaria vaccine both in the field using RTS,S malaria vaccine and in experimental setting using Whole Sporozoite malaria vaccine. We used data from three distinct studies, all conducted in Bagamoyo, Tanzania; one phase III malaria vaccine field trial, and two experimental malaria vaccine trials whose data were pooled and jointly analyzed. Bagamoyo (6.4456^0^S, 38.8989^0^E) is one of the six districts of the Pwani region located in the coastal area of Tanzania. It is bordered by the Indian ocean to the east, and is 13 meters above sea level. Bagamoyo is 8,463 km^2^ in size, with around 311,000 inhabitants in 2012 [29]. It is situated 75 Km northwest of the capital city Dar es salaam, and is one of the districts in Tanzania with a high malaria prevalence [30].

### Sources of data

#### RTS,S field study

The field trial was a phase III randomized, controlled, double-blind trial designed to investigate RTS,S malaria vaccine efficacy, safety, reactogenicity and immunogenicity in children 5–17 months and infants 6–12 weeks of age at enrolment (ClinicalTrials.gov NCT00866619). A licensed rabies vaccine or meningococcal conjugate C vaccine was administered in the children and infant control groups respectively. This was a multicenter study of up to 16,000 children enrolled from 11 trial centers covering a wide range of transmission settings in seven countries of the African Sub-Saharan region including Burkina Faso, Gabon, Ghana, Kenya, Malawi, Mozambique and Tanzania [31, 32]. The present analysis was restricted to 842 out of 1704 participants from the Bagamoyo trial center in Tanzania whose data on monocytes and lymphocytes were reported as distinct cell populations.

Screening of study participants was done after invitations and public meetings in the respective communities. Children and infants with any clinically significant acute or chronic illness, abnormal blood tests or severe malnutrition were excluded from the study. Exclusion criteria however were kept at a minimum in order to mirror the general population as far as possible while minimizing participant exposure to safety risks. Further details as inclusion and exclusion criteria are further elaborated in the original publication [31]. Vaccinations were performed between May 2009 and February 2011 during which each participant received 3 inoculations of RTS,S malaria vaccine co-administered with AS01E adjuvant, or non-malaria comparator vaccine at 1 month intervals. Clinical malaria episodes (defined as axillary temperature > = 37.5°C accompanied by >2500 *P*. *falciparum* parasites per microliter of blood in children or > = 500 *P*. *falciparum* parasites per microliter of blood in infants) were detected and recorded through passive surveillance at local health facilities within the study centers. The median of the follow-up period per child was 17.4 months.

#### BSPZV1 and BSPZV2 experimental studies

**Fig 1:**
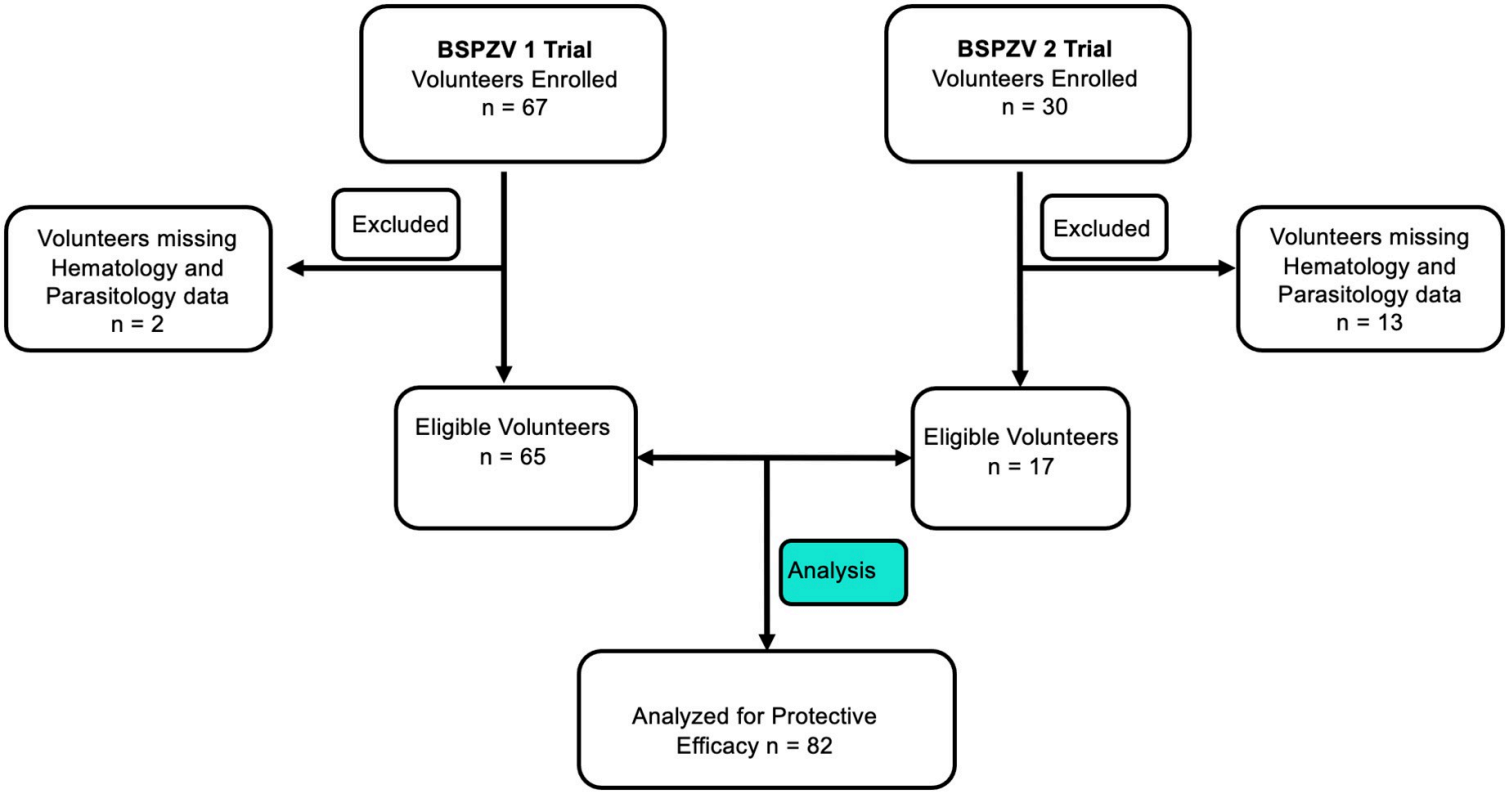
Flow chart for BSPZV1 and BSPZV2 study participants.

The first of the two experimental malaria vaccine studies whose data were pooled and jointly analyzed was known as the Bagamoyo Sporozoite Vaccine 1 (BSPZV1) trial. This was a double-blind, randomized, controlled trial conducted between April 2014 and August 2015 to assess the safety, immunogenicity and protective efficacy against controlled human malaria infection (CHMI) of whole attenuated *Plasmodium falciparum* sporozoite vaccine (pfSPZ vaccine) (ClinicalTrials.gov NCT02132299) [13]. A cohort of 67 healthy, adult (18–35 years of age) Tanzanian volunteers recruited from Bagamoyo were inoculated five times with whole, irradiation attenuated purified and cryopreserved *Plasmodium falciparum* sporozoites (pfSPZ vaccine), followed by an intravenous controlled human malaria infection (CHMI) using a homologous strain (pfSPZ challenge). A normal saline solution was administered intravenously to the control group. The volunteers in this trial were inpatients from day 9 after pfSPZ challenge injection for observation until diagnosed and treated for malaria, or until day 21 of follow-up. Over the course of follow-up, *P*.*falciparum* parasitemia was continuously monitored by the thick blood smear (TBS) approach until malaria treatment based on TBS positivity [13]. Of the 67 volunteers enrolled into the study, hematology and parasitology information was available for 65 volunteers, of whom our analysis was restricted (refer to flow chart in Fig 1).

The second experimental malaria vaccine study was the Bagamoyo Sporozoite Vaccine 2 (BSPZV2) trial. This was a double blind, randomized, placebo-controlled trial with an age de-escalation, dose escalation component to assess safety and immunogenicity of PfSPZ vaccine, and a CHMI component to assess efficacy of the vaccine (ClinicalTrials.gov NCT02613520). The trial was conducted between December 2015 and March 2017, with 30 healthy, adult Tanzanian volunteers (18–45 years of age) recruited from the Bagamoyo region constituting the CHMI component. The present analysis is restricted to the CHMI component, whose volunteers were inoculated three times with whole, irradiation attenuated purified and cryopreserved *Plasmodium falciparum* sporozoites followed by a controlled human malaria infection (CHMI) with homologous strain (pfSPZ challenge). The control group received a normal saline solution administered by direct venous inoculation. *P*. *falciparum* parasitemia was continuously monitored by TBS paired with qPCR tests. All volunteers were observed as inpatients from day 9 post CHMI until diagnosed and treated for malaria, or until day 21 of follow-up. Volunteers with positive TBS confirmed by qPCR, as well as those who were TBS negative throughout the follow-up period were treated with artemether-lumefantrine (AL) at day 28 [33]. Of the 30 volunteers enrolled in the CHMI component of the study, hematology and parasitology information was available for 17 volunteers, of whom our analysis was restricted (refer to flow chart in Fig 1).

### Ethical considerations

The RTS,S clinical trial (ClinicalTrials.gov NCT00866619) was approved by the Ifakara Health Institute Institutional Review Board (IHI-IRB), the National Health Research Ethics Review Committee (NatREC), and the Tanzania Medicines and Medical Devices Authority (TMDA), as detailed in the primary publication [31, 32]. Written or thumb-printed and witnessed informed consent was obtained from parents or legal guardians of all study participants prior to enrolment into the study.

The BSPZV1 study was approved by institutional review boards (IRBs) of the IHI (Ref. No. IHI/IRB/No:02–2014), the National Institute for Medical Research Tanzania (NIMR/HQ/R.8a/Vol.IX/1691), the Ethikkommission Nordwest-und Zentralschweiz, Basel, Switzerland (reference number 261/13), and by the Tanzania Food and Drug Authority (Ref. No. TFDA 13/CTR/0003). It was registered at Clinical Trials.gov (NCT02132299); and conducted under the U.S. Food and Drug Administration Investigational New Drug application (FDA IND) [13]. The BSPZV2 study was approved by institutional review boards (IRBs) of the Ifakara Health Institute (IHI) (Ref. No. IHI/IRB/ No: 32–2015), the National Institute for Medical Research Tanzania (NIMR/HQ/R.8a/Vol.IX/2049), and the Ethikkommission Nordwest- und Zentralschweiz, Basel, Switzerland (reference number 15/104). It was registered at ClinicalTrials.gov (NCT02613520) and approved by the Tanzania Food and Drug Authority (Auth. No. TZ15CT013), and conducted under a U.S. FDA IND application [33]. In both trials, Informed consent was obtained from all volunteers. The nature and risks of the study were explained to the volunteers, and thereafter, they were required to complete a 10-question assessment with 100% correct response rate to demonstrate understanding of the study procedures to be eligible for enrolment [13, 33].

### Assessment of monocytes and lymphocytes

In The RTS,S trial, hematology data were assessed using automated machines available at the study site in accordance to the manufacturer instructions and standard operating procedures (SOPs). Blood samples were collected in K2EDTA tubes at the study clinic and transported to the research laboratory located within the grounds of Bagamoyo district hospital. Full blood counts with differentials were assessed using Sysmex XS 800i hematology machine at baseline and multiple timepoints after vaccination. The machine provided absolute cell counts of the following parameters; white blood cells (WBC), neutrophils, basophils, eosinophils, platelets, Hemoglobin (Hb), mean corpuscular hemoglobin (MCH), mean corpuscular volume (MCV), monocytes and lymphocytes. The present analysis adopted the absolute counts of monocytes and lymphocytes to arrive at the reported ML ratios. ML ratio in this case was defined as the ratio of the absolute numbers of monocytes to lymphocytes measured from peripheral blood at baseline, before vaccination.

In both the BSPZV1 and BSPZV2 studies, T-cell immune responses were assessed using multi-parameter flow cytometry from peripheral blood mononuclear cells (PBMC) on cryopreserved samples, in a single batch, at the completion of the study [13, 34]. The flow cytometry laboratory measures collected absolute peripheral blood lymphocyte and monocyte counts as distinct cell populations, prior to either vaccination or homologous CHMI. Similar to our analysis of RTS,S trial data, we adopted the absolute counts of lymphocytes and monocytes to arrive at the reported ML ratios. ML ratio in this case was also defined as the ratio of the absolute number of monocytes to lymphocytes measured at baseline before vaccination and inoculation with homologous CHMI.

### Assessment of outcome variable

The primary outcome variable used for assessing the efficacy of RTS,S malaria vaccine was the occurrence of clinical malaria. Clinical malaria cases were detected through passive surveillance at local healthcare facilities. These cases were defined as axillary temperature > = 37.5°C accompanied by >2500 *P*. *falciparum* parasites per microliter of blood in children or > = 500 *P*. *falciparum* parasites per microliter of blood in infants. The criteria for parasitemia were assessed by thick blood smear tests. The time interval (in days) between vaccination and occurrence of first clinical malaria episode was used to analyze the effectiveness of the vaccine, and was interpreted as one minus the hazard rate (HR) (i.e. 1—HR) following a cox regression modeling.

Data from the BSPZV1 and BSPZV2 trials were pooled and jointly analyzed. The primary outcome variables of interest were the surrogate efficacy endpoints of *P*. *falciparum* parasite density, prepatent period, and multiplication rates. Parasite density was defined as the absolute number of *P*. *falciparum* parasites per microliter of blood obtained by quantitative PCR approach from peripheral blood post CHMI. Prepatent period was defined as the time interval (in days) between homologous CHMI and the first *P*. *falciparum* positive qPCR diagnosis in peripheral blood. Parasite multiplication rates (PMR) were defined as the kinetic growth rate of *P*. *falciparum* parasites in peripheral blood following homologous CHMI, and was derived from parasite density and prepatent period estimations. In both BSPZV1 and BSPZV2 trials, qPCR samples were obtained every 12 hours from day 8 to day 14 post CHMI inoculation, and then daily from day 15 to day 21 post CHMI inoculation, or until positive diagnosis by TBS test. Analyses of all qPCR samples was done retrospectively after conclusion of CHMI, except when a TBS test was positive. Vaccine efficacy was estimated by proportional analysis, and was interpreted as the proportionate reduction in incidence of developing parasitemia among the vaccinated group compared to the controls within 21 days of follow up, post CHMI.

### Statistical considerations

Participant’s characteristics including age and gender were analyzed by descriptive statistics.

Cox regression modelling was used to estimate the efficacy of RTS,S vaccine against clinical malaria. We considered possible confounding by covariates previously reported to be associated with the risk for clinical malaria, namely age, sex, distance from health facility, bed net use, and ML ratio [17, 35]. Stepwise forward variable selection procedure was used to assess and select covariates that were associated with clinical malaria, conditional on vaccination (p<0.05). Of the listed covariates, age, distance from health facility and ML ratio were included in the final regression model. In this final model, correlation between ML ratio and risk for clinical malaria was tested, whereby an interaction term between vaccination and ML ratio was added to the model to estimate the efficacy of RTS,S vaccine at different levels of baseline ML ratios. Multivariable fractional polynomial (mfp) method was used to estimate the linear and non-linear effects of ML ratio on RTS,S vaccination, but found no evidence to support the use of a model accounting for non-linearity (p = 0.64). The 842 participants available for this analysis provided more than 80% statistical power to detect a hazard rate (HR) of 0.46 of first clinical malaria episodes between the two study arms. The statistical power was calculated using *power cox* command in Stata (STATA 17 software (StataCorp LLC, College Station TX, USA).

Proportional analysis was used to estimate the efficacy of whole attenuated *Plasmodium falciparum* sporozoite vaccine in the BSPZV1 and BSPZV2 pooled dataset. Cox regression modelling was used to analyze parasite pre-patent periods, and an interaction term between ML ratio and vaccination was added to estimate the efficacy of the pfSPZ vaccine at different levels of ML ratios. Parasite multiplication rates (PMR) were derived from log-linear modeling fitted to log10-transformed quantitative PCR (qPCR) data [36, 37], and were interpreted as the coefficient of the Log-linear models for each study participant. Spearman ranks were used to assess the correlation between pre-vaccination ML ratios and protection against CHMI with respect to the surrogate endpoints of parasite density at time of qPCR diagnosis, parasite multiplication rates. We did not apply statistical procedures to account for clustering in the pooled dataset based on the assumption that since the participants were recruited from the same catchment area, received the same interventional products, were evaluated at the same trial center, and very similar approaches were followed in the studies (BSPZV1 and BSPZV2) in delivering the interventions and following up of participants, clustering effects between the experimental studies would be minimal. All analyses were performed in R software version 3.5.1. p<0.05 were considered statistically significant.

## Results

### Demographic and clinical characteristics: RTS,S vaccine trial

Demographic and clinical characteristics of participants in the RTS,S trial are tabulated in Table 1. A total of 1704 children and infants were randomly assigned to the RTS,S and control arms in the original phase III study. However, pre-vaccination monocytes and lymphocytes reported as distinct cell populations were only available for 842 children and infants, who were not significantly different from infants and children excluded from the study with respect to the demographic characteristics. As tabulated in Table 1, the baseline ML ratio did not differ significantly between vaccinated and control arms. As expected, there was a significant difference in the clinical outcomes between the vaccinated and control arms.

**Table 1:** Demographic and clinical characteristics of participants enrolled in the RTS,S trial.

Variables	Vaccinated Arm	Control Arm	P value
	(n = 557)	(n = 285)	
**Demographic**			
Median age in months (IQR)	5.0 (1.0–9.0)	5.0 (1.0–10.0)	
Sex			
Females (%)	281 (50)	138 (48)	
Distance from health facility			
< = 5Km (%)	482 (87)	256 (90)	
Bed net users (%)	485 (87)	250 (87)	
**Laboratory**	Median (IQR)	Median (IQR)	
Monocytes (μ/L)	0.8 (0.0–1.2)	0.8 (0.0–1.2)	
Lymphocytes (μ/L)	5.9 (4.6–7.5)	5.9 (4.8–7.4)	
M:L ratio	0.17 (0.0–0.2)	0.13 (0.0–0.2)	
**Clinical**			
Number of Days to First Malaria Episode	594 (569–602)	494 (412–601)	<0.05
Number of Clinical Malaria Episodes (%)	76 (13)	76 (27)	<0.05

### Statistical interaction between ML ratio and vaccination status in the RTS,S trial

In an unadjusted Cox regression model, the crude efficacy of RTS,S vaccine against the primary endpoint of time to first clinical malaria episode was 54% (95% Confidence Interval (95%CI):37%-66%, p <0.001). ML ratio, age and distance from health facility confounded the relationship between vaccination and risk for clinical malaria and contributed to model fit. Sex and bed net use were not related nor confounding and were removed in the final model. There was a negative correlation between ML ratio and risk for clinical malaria (HR:0.24, 95%CI:0.04–1.55; p = 0.02). However, when stratified by exposure status to the vaccine, ML ratio did not directly correlate with clinical malaria risk in either the vaccinated arm (HR = 0.52, 95% CI 0.48–3.13, P = 0.3) or the control arm (HR = 1.022, 95% CI 0.28–3.38, P = 0.9). The test for interaction between ML ratio and vaccination in the final multivariate model did not reach statistical significance (adjusted Hazard Rate (adjHR):0.90, 95%CI 0.45–1.80; p = 0.77). When stratified by vaccination status, the adjHR in a model with interaction was 0.80 (95%CI 0.47–1.39) for the vaccinated group and 1.14 (95%CI 0.67–1.84) for the control group.

### Demographic and laboratory characteristics: Bagamoyo sporozoite vaccine (BSPZV) trials

Data from 65 participants of the BSPZV1 trial and 17 participants of the BSPZV2 trial were appended and jointly analyzed. 91% of participants in the pooled dataset were males, and the median age in years was 24 (IQR: 22–24). Baseline ML ratio did not differ significantly between vaccinated and control arms. As expected, there was a significant difference between the groups with respect to the surrogate endpoints at first qPCR diagnosis. Demographic and laboratory characteristics in the vaccinated and control arms are further elaborated in Table 2.

**Table 2:** Demographic and laboratory characteristics of participants in the pooled BSPZV trials.

Variables	Vaccinated Arm	Control Arm	P Value
	(n = 57)	(n = 25)	
**Demographic**			
Median age in years (IQR)	24 (22–26)	25 (22–28)	
Sex			
Males (%)	53 (93)	22 (88)	
Maximum follow-up time (days)	21	14	<0.05
**Laboratory**	Median (IQR)	Median (IQR)	
Parasite Density (/μL)	0.23 (0.07–0.53)	0.35 (0.13–0.62)	<0.05
Parasite Prepatent Period (days)	9.3 (8.9–13.6)	8.5 (8.0–8.9)	<0.05
Parasite Multiplication Rates	5.2 (3.7–6.3)	5.1 (4.1–6.7)	
Monocytes	51 (42–59)	52 (44–66)	
Lymphocytes	212 (184–241)	219 (187–245)	
M:L Ratio	0.22 (0.20–0.28)	0.24 (0.21–0.28)	
qPCR -Positive Diagnosis (%)	44 (77)	25(100)	

#### Correlation between ML ratio and protection against homologous CHMI

In the pooled dataset for BSPZV1 and BSPZV2, the unadjusted efficacy of whole Sporozoite malaria vaccine by proportional analysis was 22.7% (95%CI 11.4% to 36.5%, p<0.001). In the Cox regression model, the correlation between ML ratio and parasite prepatent period did not reach statistical significance (HR = 1.16; 95%CI: 0.51–2.62, p = 0.72), and it did not interact with vaccine efficacy (p = 0.36). Furthermore, the correlation between ML ratio and either parasite density at time of qPCR diagnosis or PMR did not reach statistical significance (Spearman rho = 0.004, p = 0.97) and (Spearman rho = 0.031, p = 0.80) respectively (Table 3).

**Table 3:** Correlations between ML ratio and protection against homologous CHMI.

Correlation	Estimates
**ML ratio and Parasite Density**	Spearman rho = 0.004, p = 0.97
**ML ratio and Parasite Multiplication Rates**	Spearman rho = 0.031, p = 0.80
**ML ratio and Pre-patent Period**	Hazard Rate = 1.16; 95% CI 0.51 to 2.62; p = 0.72
**Interaction term between ML ratio vaccine**	
**efficacy**	p = 0.36

## Discussion

We investigated pre-vaccination ML ratio as a marker for effectiveness of RTS,S malaria vaccine using the clinical malaria endpoint, and as a marker for efficacy of Whole Sporozoite malaria vaccine using the surrogate endpoints. In our analysis, we could not demonstrate that the protective effect of RTS,S malaria vaccine is modified by baseline levels of ML ratios, evidenced by a non-significant statistical interaction between ML ratio and RTS,S vaccination in a Cox regression model (p = 0.77), even after stratifying ML ratios by age groups: infants (p = 0.67) and children (p = 0.59). Consistent with observations for vaccine effectiveness, we found no association between baseline ML ratios and vaccine efficacy, evidenced by a non-significant statistical interaction between ML ratio and vaccination (p = 0.36), and the absence of statistical correlations between ML ratio and the surrogate endpoints of parasite density at time of PCR diagnosis and parasite multiplication rates.

A total of 1704 children were randomly assigned to the RTS,S group or the control group. Of these, pre-vaccination ML ratios were available for 842 children (557 in the RTS,S group and 285 in the control group), of whom our analysis is restricted. The median age of the participants was 5 months at the time of vaccination. 76 clinical malaria episodes were reported in the RTS,S group and the control group (Table 1). The unadjusted efficacy of RTS,S vaccine against time to the first clinical malaria episode was 54% (95% CI 37% to 66%, p <0.001). We found a negative but insignificant correlation between ML ratio and the risk for clinical malaria (HR:0.24, 95%CI:0.04–1.55; p = 0.02). This observation is contrary to the study by Warimwe et al, 2013 [18], which reports a positive correlation between ML ratio and the risk for clinical malaria, but only for individuals who were parasite-positive at baseline [18]. When stratified by vaccination status, our observations are consistent with the report by Warimwe et al, 2013 [17], in which ML ratio did not directly correlate with the risk for clinical malaria either in the vaccinated arm (HR = 1.022, 95% CI 0.28–3.38, P = 0.9) or the control arm (HR = 0.52, 95% CI 0.48–3.13, P = 0.3). Contrary to the observations by Warimwe et al 2013 [17] that reported for the first time that pre-vaccination ML ratio accounts for the variation in the effectiveness of RTS,S malaria vaccine, we did not find an association between ML ratio and the efficacy of RTS,S vaccine, based on a non-significant interaction term between pre-vaccination ML ratios and RTS,S vaccination in a Cox regression model.

Despite the similarities between the current RTS,S field study and that previously reported by Warimwe et.al, 2013 [17], there are notable differences that one should take into account when interpreting the reports. Firstly, there is a possibility for underlying differences between the study populations with respect to levels of malaria parasite exposure, and consequently, a differential impact on monocyte characteristics and function [17, 35]. At the time the studies were conducted, the relative measure of intensity of malaria exposure was low to moderate in Kenya and Gabon respectively, where data for the study by Warimwe et.al 2013 were collected, while in Bagamoyo, Tanzania, it was heterogeneous [38]. Warimwe et.al, 2013 [17], accounted for parasite exposure index on a small proportion of the overall population analyzed, while in the present study, we did not have means to assess the parasite exposures. Nevertheless, we believe that these variances must have been taken care-of with randomization, and thus do not compromise our estimations. Another difference is the fact that both studies did not account for baseline asymptomatic malaria infection status in the trial participants. Pre-clinical malaria infection highly influences changes in leukocyte numbers and morphology, and is often accompanied with significant monocytosis and lymphocytopenia [39–42]. We therefore hypothesize that asymptomatic malaria before and in-between vaccination doses might have impacted the ML ratios and thus influenced the risk for clinical malaria, regardless of vaccination. Again, we believe that due to randomization, possible differences between the groups with respect to the baseline variables are minimal.

In addition to the RTS,S field trial, our investigation also analyzed the relationship between ML ratio and PfSPZ vaccine efficacy in an experimental setting following a CHMI model. To the best of our knowledge, this is the first investigation of the relationship between peripheral blood ML ratio and the efficacy of a malaria vaccine within the context of a CHMI model. A CHMI model offers unique advantages over a field trial. Firstly, it offers a possibility for defining the precise number of sporozoites each trial participant is exposed to, thus enabling trialists to standardize parasite inoculum dosages between study groups [13, 33, 43, 44]. Secondly, in a CHMI model, trial participants are inoculated with sporozoites at defined time points, which allows for more accurate estimations of associations between parasite exposure and immune responses to malaria [45]. Finally, the advent use of quantitative PCR in a CHMI model facilitates the monitoring of a malaria infection from the lowest end of the spectrum of the infection before onset of clinical symptoms, through the use of surrogate endpoints of malaria parasite density, pre-patent period and multiplication rates. The use of surrogate endpoints enables the assessment of parameters within the causal pathway of a malaria infection, and thus facilitates a more objective assessment of the relationship between malaria infection and immune responses from a biological point of view.

A total of 97 volunteers were randomly assigned to PfSPZ vaccine group or the control group in the original BSPZV1 and BSPZV2 studies. Of these, pre-vaccination ML ratios were available for 82 participants, of whom our analysis is restricted (Fig 1). As tabulated in Table 2, the median age at the time of vaccination was 25 years, and the maximum follow-up time before malaria diagnosis was 21 days in the PfSPZ arm and 14 days in the control arm. There was a significant difference between the PfSPZ and control arms with respect to parasite density and prepatent period (Table 2), but the unadjusted efficacy of PfSPZ vaccine by proportional analysis was 17.6% (95%CI 10% to 28.5%, p<0.001). Consistent with the observations of the RTS,S field trial reported in this study, we report no association between pre-vaccination ML ratios and the efficacy of PfSPZ malaria vaccine in context of experimental malaria vaccine study followed by homologous CHMI. In our analysis, baseline ML ratio negatively correlated with the three surrogate endpoints, but did not reach statistical significance. There was no evidence for a statistical interaction between ML ratio and PfSPZ vaccine efficacy (Table 3). We do not yet have a proven explanation for the lack of association, but we hypothesize several reasons. The behavior and functionalities of monocytes may be affected by many factors such as age, history of malaria exposure, and malaria transmission intensity [46–49]. Moreover, monocyte-to-lymphocyte ratio, which is based on the absolute numbers of monocytes and lymphocytes, by itself, may not be a sensitive enough marker to reflect the underlying cellular interactions that may equally be important in conferring protection upon vaccination. This has been made evident in studies that report interactions between monocytes and gamma-delta (γδ) T-cells that are important in conferring protection upon infection with malaria parasites [50]. For these reasons, ML ratios alone may not be an adequate marker for predicting protection or refractory to protection with immunization by malaria vaccines.

We acknowledge several limitations in the analysis of experimental malaria vaccine trial followed by homologous CHMI. Firstly, although statistically significant, the efficacy of pfSPZ vaccine in the combined dataset was relatively low, at 17.6% (95%CI: 10%-28.5%, P<0.001). Since the low efficacy implies that the difference between the groups with respect to the outcome variable was only slight, it is difficult to identify factors responsible for the differences, unless such factors have very large effect size. Therefore, the low efficacy of the vaccine, although significant, might have missed the detection of possible marginal influences of ML ratio on efficacy of the vaccine. Secondly, the CHMI trials were performed in adult participants who have been repeatedly exposed to malaria parasites and thus have developed semi-immunity to clinical malaria [51]. Modulation of parasite dynamics is in some way influenced by mechanisms of semi immunity, which then perhaps makes ML ratios per se not an important marker for tracing progression of an infection [52].

It is important to note that studies investigating the role of monocytes in malaria infections need to be interpreted with caution. Monocytes are a heterogeneous population of cells whose behavior and functionalities are shaped by many factors, as pointed out earlier. Because of this, contradictory observations of monocyte behavior across studies are not uncommon, independent of study protocols and analytic techniques [53]. Furthermore, our reports on subgroup analyses are based on non-significant interaction terms in regression models. It is important to note that non-significant statistical interactions might not always reflect absence of a biologic interaction, but rather may merely suggest that the functional form of the mathematical model is not a proper description of the relation between the variables. In the present analysis, this is evidenced by the notable differences in the strata-specific adjusted hazard rates in the vaccinated arm (aHR = 0.80, 95%CI 0.47–1.39) compared to the control arm (aHR = 1.14, 95%CI 0.67–1.84).

## Conclusion

We could not demonstrate that the variation in efficacy of malaria vaccines between recipients is attributed to differences in pre-vaccination monocyte-to-lymphocyte ratios. Our observations have been consistent in both RTS,S subunit vaccine administered in children in a field study and in Attenuated Whole Sporozoite malaria vaccine administered in adults in experimental malaria vaccine study followed by controlled human malaria infection. It appears that monocyte-to-lymphocyte ratio is not an adequate independent marker for protection or refractory to protection with immunization by malaria vaccines. Further investigations on immune correlates of protection are important in context of malaria vaccination as it will inform what to measure in individuals to tell if they will be protected or not protected by vaccination. Furthermore, Molecular investigations of underlying mechanisms of protected immunity are important as they could provide a clearer explanation of the differences between those protected in comparison with those not protected.

## Supporting information

S1 Data (ZIP)
