# Supplementary material for: Pre-vaccination monocyte-to-lymphocyte ratio as a biomarker for the efficacy of malaria candidate vaccines: A subgroup analysis of pooled clinical trial data
Source: PLoS One. 2023 Sep 14;18(9):e0291244. doi: 10.1371/journal.pone.0291244 (PMC10501550; doi:10.1371/journal.pone.0291244)
Supplement: S1 Data — (ZIP) [file pone.0291244.s001.zip › Manuscript Data/Database_Documentation.doc]

Study MALARIA-055 PRI (110021)

| **Dataset** | **Description of dataset** | **Keys** |
| --- | --- | --- |
| Bednet | Bednet and spraying | PID, ACTIVITY. |
| codelist | List codes and labels by coded variables | CODELIST, CODE |
| efficacy | Clinical malaria events. | PID, DATE. |
| Expogn | Study vaccination | PID, RELVACDO, P_CODE |
| fueeimputed | Follow up time per pid. | PID. |
| orgalladmittee | Cultured organisms at admission for ITT until end extension | PID, DATE |
| Pid | List of subj with group / cohort status | PID |
| serocod | Immunogenicity (serology results) | PID, ACTIVITY, LAB_CODE, SEROTEST |
| sevmal | Severe malaria and other serious illness events. | PID, DATE. |
| wconvac | Concomitant vaccination | PID, TRADNAME, MEDSRDAT |
| Wdates | Dates of visits and DB activity numbers. | PID, ACTIVITY, TYPE_LST |
| wdemog | Demography | PID, ACTIVITY |
| Wgrow | Growth parameters. | PID, ACTIVITY. |
| wicd10 | ICD-10 codes and decodes (including Verbal Autopsies) | PID |
| wmedic | Medication | PID, TRADNAME, MEDSRDAT |
| wmortal | All cause mortality | PID |
| wunsol | Unsolicited adverse events | PID, AE_DESC, AE_SRDAT, P_CODE, AE_INTEN |

Study 110021 - bednet

| **Variable** | **Description** | **Type** | **Codes** | **Crf Page** | **Comment** |
| --- | --- | --- | --- | --- | --- |
| ACTIVITY | Activity | Num 8 |  |  | From *.activity field on top of first page of each CRF segment |
| AGEC | Age Group (N) | Num 8 | 1 = [5-17] months |  | Derived: if 5<=age<= 17 months then AGEC=1; if 6<=age<= 12 weeks then AGEC=2 |
|  |  |  | 2 = [6-12] weeks |  |  |
| AGE_LABEL | Age Group | Char 13 |  |  | Derived: if 5<=age<= 17 months then AGE_LABEL=”[5-17] months”; if 6<=age<= 12 weeks then AGE_LABEL=”[6-12] weeks” |
| BEDNT | Bed-net | Char 6 |  |  | Derived: if BEDNT_YN equals 1, 4 or 5 then BEDNT=”No ITN”, if BEDNT_YN equals 2 or 3 then BEDNT=”ITN”, Null otherwise |
|  |  |  | ITN |  |  |
|  |  |  | No ITN |  |  |
| BEDNT_YN | Sleeping under a bed net? | Char 3 |  | crf1:82, 123, 144 | bednet.bednt_yn |
|  |  |  | 1 = NO |  |  |
|  |  |  | 2 = IMPREGNATED BED NET NO HOLES |  |  |
|  |  |  | 2 = IMPREGNATED BED NET WITH NO HOLE LARGE ENOUGH TO ADMIT THREE FINGERS |  |  |
|  |  |  | 3 = IMPREGNATED BED NET HOLES |  |  |
|  |  |  | 3 = IMPREGNATED BED NET WITH AT LEAST ONE HOLE LARGE ENOUGH TO ADMIT THREE FINGERS |  |  |
|  |  |  | 4 = UNTREATED BED NET NO HOLES |  |  |
|  |  |  | 4 = UNTREATED BED NET WITH NO HOLE LARGE ENOUGH TO ADMIT THREE FINGERS |  |  |
|  |  |  | 5 = UNTREATED BED NET HOLES |  |  |
|  |  |  | 5 = UNTREATED BED NET WITH AT LEAST ONE HOLE LARGE ENOUGH TO ADMIT THREE FINGERS |  |  |
|  |  |  | MC = MISSING CONFIRMED |  |  |
| CENTER | Center number | Char 10 |  |  | See pid.center |
| CTY_COD | Country code | Char 3 | BF = Burkina Faso |  | ctr_info.cty_cod |
|  |  |  | GA = Gabon |  |  |
|  |  |  | GH = Ghana |  |  |
|  |  |  | KE = Kenya |  |  |
|  |  |  | MW = Malawi |  |  |
|  |  |  | MZ = Mozambique |  |  |
|  |  |  | TZ = Tanzania |  |  |
| CTY_NAM | Country name | Char 30 | Burkina Faso |  | ctr_info.cty_nam |
|  |  |  | Gabon |  |  |
|  |  |  | Ghana |  |  |
|  |  |  | Kenya |  |  |
|  |  |  | Malawi |  |  |
|  |  |  | Mozambique |  |  |
|  |  |  | Tanzania |  |  |
| ELIG_CE | Eligibility(CE) | Char 1 | E = subject eliminated from the ATP cohort for safety and the ATP cohort for immunogenicity for elimination type |  | See Elim codes overview |
|  |  |  | I = subject eliminated from the ATP cohort for immunogenicity but not from the ATP cohort for safety for elimination type |  |  |
| ELIG_CM | Eligibility(CM) | Char 1 | E = subject eliminated from the ATP cohort for safety and the ATP cohort for immunogenicity for elimination type |  |  |
|  |  |  | I = subject eliminated from the ATP cohort for immunogenicity but not from the ATP cohort for safety for elimination type |  |  |
| ELIG_HS | Eligibility(HS) | Char 1 | I = subject eliminated from the ATP cohort for immunogenicity but not from the ATP cohort for safety for elimination type |  |  |
| ELIG_HV | Eligibility(HV) | Char 1 | E = subject eliminated from the ATP cohort for safety and the ATP cohort for immunogenicity for elimination type |  |  |
|  |  |  | I = subject eliminated from the ATP cohort for immunogenicity but not from the ATP cohort for safety for elimination type |  |  |
| ELIG_IE | Eligibility(IE) | Char 1 | E = subject eliminated from the ATP cohort for safety and the ATP cohort for immunogenicity for elimination type |  |  |
|  |  |  | I = subject eliminated from the ATP cohort for immunogenicity but not from the ATP cohort for safety for elimination type |  |  |
| ELIG_IM | Eligibility(IM) | Char 1 | E = subject eliminated from the ATP cohort for safety and the ATP cohort for immunogenicity for elimination type |  |  |
|  |  |  | I = subject eliminated from the ATP cohort for immunogenicity but not from the ATP cohort for safety for elimination type |  |  |
| ELIG_PT | Eligibility(PT) | Char 1 | E = subject eliminated from the ATP cohort for safety and the ATP cohort for immunogenicity for elimination type |  |  |
|  |  |  | I = subject eliminated from the ATP cohort for immunogenicity but not from the ATP cohort for safety for elimination type |  |  |
| ELIG_RI | Eligibility(RI) | Char 1 | E = subject eliminated from the ATP cohort for safety and the ATP cohort for immunogenicity for elimination type |  |  |
|  |  |  | I = subject eliminated from the ATP cohort for immunogenicity but not from the ATP cohort for safety for elimination type |  |  |
| ELIG_SM | Eligibility(SM) | Char 1 | E = subject eliminated from the ATP cohort for safety and the ATP cohort for immunogenicity for elimination type |  |  |
|  |  |  | I = subject eliminated from the ATP cohort for immunogenicity but not from the ATP cohort for safety for elimination type |  |  |
| ELIM_RCE | Elim reacto(CE) | Num 8 | 1 = subject eliminated from the ATP cohort for safety for elimination type |  |  |
| ELIM_RCM | Elim reacto(CM) | Num 8 | 1 = subject eliminated from the ATP cohort for safety for elimination type |  |  |
| ELIM_RHS | Elim reacto(HS) | Num 8 |  |  |  |
| ELIM_RHV | Elim reacto(HV) | Num 8 | 1 = subject eliminated from the ATP cohort for safety for elimination type |  |  |
| ELIM_RIE | Elim reacto(IE) | Num 8 | 1 = subject eliminated from the ATP cohort for safety for elimination type |  |  |
| ELIM_RIM | Elim reacto(IM) | Num 8 | 1 = subject eliminated from the ATP cohort for safety for elimination type |  |  |
| ELIM_RPT | Elim reacto(PT) | Num 8 | 1 = subject eliminated from the ATP cohort for safety for elimination type |  |  |
| ELIM_RRI | Elim reacto(RI) | Num 8 | 1 = subject eliminated from the ATP cohort for safety for elimination type |  |  |
| ELIM_RSM | Elim reacto(SM) | Num 8 | 1 = subject eliminated from the ATP cohort for safety for elimination type |  |  |
| ELIM_SCE | Elim sero(CE) | Num 8 | 1 = subject eliminated from the ATP cohort for immunogenicity for elimination type |  |  |
| ELIM_SCM | Elim sero(CM) | Num 8 | 1 = subject eliminated from the ATP cohort for immunogenicity for elimination type |  |  |
| ELIM_SHS | Elim sero(HS) | Num 8 | 1 = subject eliminated from the ATP cohort for immunogenicity for elimination type |  |  |
| ELIM_SHV | Elim sero(HV) | Num 8 | 1 = subject eliminated from the ATP cohort for immunogenicity for elimination type |  |  |
| ELIM_SIE | Elim sero(IE) | Num 8 | 1 = subject eliminated from the ATP cohort for immunogenicity for elimination type |  |  |
| ELIM_SIM | Elim sero(IM) | Num 8 | 1 = subject eliminated from the ATP cohort for immunogenicity for elimination type |  |  |
| ELIM_SPT | Elim sero(PT) | Num 8 | 1 = subject eliminated from the ATP cohort for immunogenicity for elimination type |  |  |
| ELIM_SRI | Elim sero(RI) | Num 8 | 1 = subject eliminated from the ATP cohort for immunogenicity for elimination type |  |  |
| ELIM_SSM | Elim sero(SM) | Num 8 | 1 = subject eliminated from the ATP cohort for immunogenicity for elimination type |  |  |
| ELIM_ST | Elim Stat | Num 8 |  |  |  |
| ELIM_V | Elim Vaccin | Num 8 |  |  |  |
| ELI_F3CE | Elim Efficacy(CE/3) | Num 8 | 1 = subject eliminated from the ATP population for efficacy for elimination type |  |  |
| ELI_F3CM | Elim Efficacy(CM/3) | Num 8 | 1 = subject eliminated from the ATP population for efficacy for elimination type |  |  |
| ELI_F3HS | Elim Efficacy(HS/3) | Num 8 |  |  |  |
| ELI_F3HV | Elim Efficacy(HV/3) | Num 8 | 1 = subject eliminated from the ATP population for efficacy for elimination type |  |  |
| ELI_F3IE | Elim Efficacy(IE/3) | Num 8 | 1 = subject eliminated from the ATP population for efficacy for elimination type |  |  |
| ELI_F3IM | Elim Efficacy(IM/3) | Num 8 | 1 = subject eliminated from the ATP population for efficacy for elimination type |  |  |
| ELI_F3PT | Elim Efficacy(PT/3) | Num 8 | 1 = subject eliminated from the ATP population for efficacy for elimination type |  |  |
| ELI_F3RI | Elim Efficacy(RI/3) | Num 8 | 1 = subject eliminated from the ATP population for efficacy for elimination type |  |  |
| ELI_F3SM | Elim Efficacy(SM/3) | Num 8 | 1 = subject eliminated from the ATP population for efficacy for elimination type |  |  |
| ELI_F4CE | Elim Efficacy(CE/4) | Num 8 | 1 = subject eliminated from the ITT population for elimination type |  |  |
| ELI_F4CM | Elim Efficacy(CM/4) | Num 8 |  |  |  |
| ELI_F4HS | Elim Efficacy(HS/4) | Num 8 | 1 = subject eliminated from the ITT population for elimination type |  |  |
| ELI_F4HV | Elim Efficacy(HV/4) | Num 8 |  |  |  |
| ELI_F4IE | Elim Efficacy(IE/4) | Num 8 | 1 = subject eliminated from the ITT population for elimination type |  |  |
| ELI_F4IM | Elim Efficacy(IM/4) | Num 8 |  |  |  |
| ELI_F4PT | Elim Efficacy(PT/4) | Num 8 |  |  |  |
| ELI_F4RI | Elim Efficacy(RI/4) | Num 8 |  |  |  |
| ELI_F4SM | Elim Efficacy(SM/4) | Num 8 |  |  |  |
| ELI_F5CE | Elim Efficacy(CE/5) | Num 8 | 1 = subject eliminated from the ATP population for efficacy for elimination type |  |  |
| ELI_F5CM | Elim Efficacy(CM/5) | Num 8 | 1 = subject eliminated from the ATP population for efficacy for elimination type |  |  |
| ELI_F5HS | Elim Efficacy(HS/5) | Num 8 | 1 = subject eliminated from the ATP population for efficacy for elimination type |  |  |
| ELI_F5HV | Elim Efficacy(HV/5) | Num 8 | 1 = subject eliminated from the ATP population for efficacy for elimination type |  |  |
| ELI_F5IE | Elim Efficacy(IE/5) | Num 8 | 1 = subject eliminated from the ATP population for efficacy for elimination type |  |  |
| ELI_F5IM | Elim Efficacy(IM/5) | Num 8 | 1 = subject eliminated from the ATP population for efficacy for elimination type |  |  |
| ELI_F5PT | Elim Efficacy(PT/5) | Num 8 | 1 = subject eliminated from the ATP population for efficacy for elimination type |  |  |
| ELI_F5RI | Elim Efficacy(RI/5) | Num 8 | 1 = subject eliminated from the ATP population for efficacy for elimination type |  |  |
| ELI_F5SM | Elim Efficacy(SM/5) | Num 8 | 1 = subject eliminated from the ATP population for efficacy for elimination type |  |  |
| F2200CAD | F2200CAD | Char 1 | N = No |  | Infant belongs to the immune subset |
|  |  |  | Y = Yes |  |  |
| F2200OLD | F2200OLD | Char 1 | N = No |  | Child belongs to the immune subset |
|  |  |  | Y = Yes |  |  |
| F6000OLD | F6000OLD | Char 1 | N = No |  | pid.belongs to the first 6000 enrolled |
|  |  |  | Y = Yes |  |  |
| GROUP_NB | Group number | Num 8 | 1 = R3R |  | See pid.group_nb |
|  |  |  | 2 = R3C |  |  |
|  |  |  | 3 = C3C |  |  |
| INSTR_YN | Residual spray used? | Char 3 | N = No | crf1:82, 123, 144 | bednet.instr_yn |
|  |  |  | Y = Yes |  |  |
| PID | Subject Identification | Num 8 |  |  | From *.pid field on the first page of the CRF |
| RND_ID | Treatment number | Num 8 |  |  | See pid.rnd_id |
| SCHEDULE | Schedule (clinical effective) | Num 8 |  |  | See pid.schedule |
| SITE | Site | Char 9 | Agogo |  | See pid.site |
|  |  |  | Bagamoyo |  |  |
|  |  |  | Kilifi |  |  |
|  |  |  | Kintampo |  |  |
|  |  |  | Kombewa |  |  |
|  |  |  | Korogwe |  |  |
|  |  |  | Lambarene |  |  |
|  |  |  | Lilongwe |  |  |
|  |  |  | Manhica |  |  |
|  |  |  | Nanoro |  |  |
|  |  |  | Siaya |  |  |
| TREATMNT | Treatment | Char 200 | Control |  | See pid.treatmnt |
|  |  |  | RTS,S/AS01E primary schedule with booster |  |  |
|  |  |  | RTS,S/AS01E primary schedule without booster |  |  |

Study 110021 - codelist

| **Variable** | **Description** | **Type** | **Codes** | **Crf Page** | **Comment** |
| --- | --- | --- | --- | --- | --- |
| CODE | Codes | Char 400 |  |  | From data management. |
| CODELIST | Name of the list of codes | Char 80 |  |  | From data management. |
| LABEL | Short label of the code | Char 400 |  |  | From data management. |
| LONGLABL | Long label of the code | Char 400 |  |  | From data management. |
| VALUE | Values corresponding to the code | Char 400 |  |  | From data management. Same as (code). |

Study 110021 - efficacy

| **Variable** | **Description** | **Type** | **Codes** | **Crf Page** | **Comment** |
| --- | --- | --- | --- | --- | --- |
| BSL_DAT | Date of blood slide | Char 9 |  | crf1:207 | slide.bsl_dat  Date of blood slide, taken from dataset slide |
| DATE | Date | Num 8 |  | crf1:154 | outpat.pres_dt  Date of presentation (outpatients), converted to a numeric variable. Refer to variable INPAT to determine whether the record refers to an in- or an outpatient record. |
|  |  |  |  | crf1:184, 196 | signs.adm_dt  Date of admission (inpatients), converted to a numeric variable. Refer to variable INPAT to determine whether the record refers to an in- or an outpatient record. |
| FEVER | Fever | Char 2 | N = No | crf1:154 | outpat.fever  Depending on whether the subject was an inpatient or outpatient, fever is taken from either signs (inpatients) or outpat (outpatients). |
|  |  |  | Y = Yes | crf1:184 | symp_his.fever  Depending on whether the subject was an inpatient or outpatient, fever is taken from either signs (inpatients) or outpat (outpatients). |
| FINALDEN | Asexual P Falciparum density | Num 8 |  |  | See wslide.a_pf_den  Final parasite density |
| INPAT | Inpatient | Num 8 | 0 = No |  | Derived: Dummy variable indicating whether the record refers to an inpatient (value 1) or an outpatient (value 0). |
|  |  |  | 1 = Yes |  |  |
| PID | Subject Identification | Num 8 |  |  | From *.pid field on the first page of the CRF |
| TEMP | Temp | Num 8 |  | crf1:154 | outpat.temp_val  Depending on whether the subject was an inpatient or outpatient, temp is taken from outpat (outpatients). The value is converted to a number |
|  |  |  |  | crf1:185, 196 | signs.temp_val  Depending on whether the subject was an inpatient or outpatient, temp is taken from signs (inpatients). The value is converted to a number |

Study 110021 - expogn

| **Variable** | **Description** | **Type** | **Codes** | **Crf Page** | **Comment** |
| --- | --- | --- | --- | --- | --- |
| ACTIVITY | Activity | Num 8 | 20 = VISIT2 |  | From *.activity field on top of first page of each CRF segment |
|  |  |  | 220 = VISIT22 |  |  |
|  |  |  | 30 = VISIT3 |  |  |
|  |  |  | 40 = VISIT4 |  |  |
| GROUP_NB | Group number | Num 8 | 1 = R3R |  | See pid.group_nb |
|  |  |  | 2 = R3C |  |  |
|  |  |  | 3 = C3C |  |  |
| PID | Subject Identification | Num 8 |  |  | From *.pid field on the first page of the CRF |
| P_AP | Vacc administered according to protocol | Char 2 | N = NOT ADMINISTERED | crf1:29, 31, 41, 43, 53, 55, 95, 96 | vaccprod.p_ap |
|  |  |  | Y = ADMINISTERED ACCORDING TO PROTOCOL |  |  |
| P_CODE | Vaccine code unblinded | Char 3 |  |  | Derived: internal vaccine code after unblinding. Unblinded value is based on the blinded code (vaccprod.p_code) and the treatment group (pid.group_nb). |
| P_RDAT | Date of vaccination | Char 9 |  | crf1:1, 25, 41, 43, 53, 55, 65, 91, 107, 125, 138, 140, 146 | actdates.actrdate  (Date of vaccination with administration at a visit) |
|  |  |  |  | crf1:30, 31, 42, 43, 54, 55, 95, 96 | v_info.vaccrdat  (Date of vaccination with administration not during a visit) |
| RELVACDO | Activity related vaccine dose | Num 8 | . |  | From protocol. Vaccine dose planned at the activity in the protocol. So this dose number is directly related to the activity irrespective of whether the dose was administered or not. |
|  |  |  | 1 |  |  |
|  |  |  | 2 |  |  |
|  |  |  | 3 |  |  |
|  |  |  | 4 |  |  |
| SCHEDULE | Schedule (clinical effective) | Num 8 |  |  | See pid.schedule |
| VACCINE | Vaccine name | Char 87 |  |  | Derived: name of the administered vaccine. This is the label attached to the product code (p_code). |
|  |  |  | MENJUGATE |  |  |
|  |  |  | OPV |  |  |
|  |  |  | RTS,S/AS01E |  |  |
|  |  |  | TRITANRIX-HEPB MIXED WITH HIBERIX |  |  |
|  |  |  | VERORAB |  |  |
| VACC_ADM | Subject vaccinated? | Char 2 | N = VACCINE NOT ADMINISTERED |  | Derived: ‘Y’ when (vial_typ) = ‘S’ , ‘R’ or ‘W’. |
|  |  |  | Y = VACCINE ADMINISTERED |  |  |
| VIAL_TYP | Vaccine administration | Char 2 | N = NOT ADMINISTERED (X) | crf1:29, 31, 41, 43, 53, 55, 95, 96 | vaccprod.vial_typ |
|  |  |  | R = REPLACEMENT VIAL |  |  |
|  |  |  | S = STUDY VACCINE |  |  |
|  |  |  | W = WRONG VIAL NUMBER |  |  |
| V_ADM | Any study vaccination at visit? | Char 1 | N = no vaccination at the visit |  | Derived: ‘Y’ if vial_typ = ‘S’, ‘R’ or ‘W’ for at least one study vaccine at the visit. |
|  |  |  | Y = at least one vaccination at the visit |  |  |

Study 110021 - fueeimputed :

| **Variable** | **Description** | **Type** | **Codes** | **Crf Page** | **Comment** |
| --- | --- | --- | --- | --- | --- |
| AGEC | Age Group (N) | Num 8 | 1 = [5-17] months |  | Derived: if 5<=age<= 17 months then AGEC=1; if 6<=age<= 12 weeks then AGEC=2 |
|  |  |  | 2 = [6-12] weeks |  |  |
| AGE_LABEL | Age Group | Char 13 |  |  | Derived: if 5<=age<= 17 months then AGE_LABEL=”[5-17] months”; if 6<=age<= 12 weeks then AGE_LABEL=”[6-12] weeks” |
| CENTER | Center number | Char 10 |  |  | See pid.center |
| CTY_COD | Country code | Char 3 | BF = Burkina Faso |  | ctr_info.cty_cod |
|  |  |  | GA = Gabon |  |  |
|  |  |  | GH = Ghana |  |  |
|  |  |  | KE = Kenya |  |  |
|  |  |  | MW = Malawi |  |  |
|  |  |  | MZ = Mozambique |  |  |
|  |  |  | TZ = Tanzania |  |  |
| CTY_NAM | Country name | Char 30 | Burkina Faso |  | ctr_info.cty_nam |
|  |  |  | Gabon |  |  |
|  |  |  | Ghana |  |  |
|  |  |  | Kenya |  |  |
|  |  |  | Malawi |  |  |
|  |  |  | Mozambique |  |  |
|  |  |  | Tanzania |  |  |
| ELIG_CE | Eligibility(CE) | Char 1 | E = subject eliminated from the ATP cohort for safety and the ATP cohort for immunogenicity for elimination type |  | See pid.elig_ce |
|  |  |  | I = subject eliminated from the ATP cohort for immunogenicity but not from the ATP cohort for safety for elimination type |  |  |
| ELIG_CM | Eligibility(CM) | Char 1 | E = subject eliminated from the ATP cohort for safety and the ATP cohort for immunogenicity for elimination type |  | See Elim codes overview |
|  |  |  | I = subject eliminated from the ATP cohort for immunogenicity but not from the ATP cohort for safety for elimination type |  |  |
| ELIG_HS | Eligibility(HS) | Char 1 | I = subject eliminated from the ATP cohort for immunogenicity but not from the ATP cohort for safety for elimination type |  |  |
| ELIG_HV | Eligibility(HV) | Char 1 | E = subject eliminated from the ATP cohort for safety and the ATP cohort for immunogenicity for elimination type |  |  |
|  |  |  | I = subject eliminated from the ATP cohort for immunogenicity but not from the ATP cohort for safety for elimination type |  |  |
| ELIG_IE | Eligibility(IE) | Char 1 | E = subject eliminated from the ATP cohort for safety and the ATP cohort for immunogenicity for elimination type |  |  |
|  |  |  | I = subject eliminated from the ATP cohort for immunogenicity but not from the ATP cohort for safety for elimination type |  |  |
| ELIG_IM | Eligibility(IM) | Char 1 | E = subject eliminated from the ATP cohort for safety and the ATP cohort for immunogenicity for elimination type |  |  |
|  |  |  | I = subject eliminated from the ATP cohort for immunogenicity but not from the ATP cohort for safety for elimination type |  |  |
| ELIG_PT | Eligibility(PT) | Char 1 | E = subject eliminated from the ATP cohort for safety and the ATP cohort for immunogenicity for elimination type |  |  |
|  |  |  | I = subject eliminated from the ATP cohort for immunogenicity but not from the ATP cohort for safety for elimination type |  |  |
| ELIG_RI | Eligibility(RI) | Char 1 | E = subject eliminated from the ATP cohort for safety and the ATP cohort for immunogenicity for elimination type |  |  |
|  |  |  | I = subject eliminated from the ATP cohort for immunogenicity but not from the ATP cohort for safety for elimination type |  |  |
| ELIG_SM | Eligibility(SM) | Char 1 | E = subject eliminated from the ATP cohort for safety and the ATP cohort for immunogenicity for elimination type |  |  |
|  |  |  | I = subject eliminated from the ATP cohort for immunogenicity but not from the ATP cohort for safety for elimination type |  |  |
| ELIM_ATB | Elim (ATB) | Num 8 | . |  |  |
|  |  |  | 1 |  | Eliminated from ATP analyses starting at time of booster dose |
| ELIM_ATP | Elim (ATP) | Num 8 | . |  |  |
|  |  |  | 1 |  | Eliminated from ATP analyses starting at 14 days post dose 3 |
| ELIM_ITB | Elim (ITB) | Num 8 | . |  |  |
|  |  |  | 1 |  | Eliminated from ITT analyses starting at time of booster dose |
| ELIM_ITT | Elim (ITT) | Num 8 |  |  | Eliminated from ITT analyses starting at dose 1 |
| ELIM_RCE | Elim reacto(CE) | Num 8 | 1 = subject eliminated from the ATP cohort for safety for elimination type |  | See Elim codes overview |
| ELIM_RCM | Elim reacto(CM) | Num 8 | 1 = subject eliminated from the ATP cohort for safety for elimination type |  |  |
| ELIM_RHS | Elim reacto(HS) | Num 8 |  |  |  |
| ELIM_RHV | Elim reacto(HV) | Num 8 | 1 = subject eliminated from the ATP cohort for safety for elimination type |  |  |
| ELIM_RIE | Elim reacto(IE) | Num 8 | 1 = subject eliminated from the ATP cohort for safety for elimination type |  |  |
| ELIM_RIM | Elim reacto(IM) | Num 8 | 1 = subject eliminated from the ATP cohort for safety for elimination type |  |  |
| ELIM_RPT | Elim reacto(PT) | Num 8 | 1 = subject eliminated from the ATP cohort for safety for elimination type |  |  |
| ELIM_RRI | Elim reacto(RI) | Num 8 | 1 = subject eliminated from the ATP cohort for safety for elimination type |  |  |
| ELIM_RSM | Elim reacto(SM) | Num 8 | 1 = subject eliminated from the ATP cohort for safety for elimination type |  |  |
| ELIM_SCE | Elim sero(CE) | Num 8 | 1 = subject eliminated from the ATP cohort for immunogenicity for elimination type |  |  |
| ELIM_SCM | Elim sero(CM) | Num 8 | 1 = subject eliminated from the ATP cohort for immunogenicity for elimination type |  |  |
| ELIM_SHS | Elim sero(HS) | Num 8 | 1 = subject eliminated from the ATP cohort for immunogenicity for elimination type |  |  |
| ELIM_SHV | Elim sero(HV) | Num 8 | 1 = subject eliminated from the ATP cohort for immunogenicity for elimination type |  |  |
| ELIM_SIE | Elim sero(IE) | Num 8 | 1 = subject eliminated from the ATP cohort for immunogenicity for elimination type |  |  |
| ELIM_SIM | Elim sero(IM) | Num 8 | 1 = subject eliminated from the ATP cohort for immunogenicity for elimination type |  |  |
| ELIM_SPT | Elim sero(PT) | Num 8 | 1 = subject eliminated from the ATP cohort for immunogenicity for elimination type |  |  |
| ELIM_SRI | Elim sero(RI) | Num 8 | 1 = subject eliminated from the ATP cohort for immunogenicity for elimination type |  |  |
| ELIM_SSM | Elim sero(SM) | Num 8 | 1 = subject eliminated from the ATP cohort for immunogenicity for elimination type |  |  |
| ELIM_ST | Elim Stat | Num 8 |  |  |  |
| ELIM_V | Elim Vaccin | Num 8 |  |  |  |
| ELI_F3CE | Elim Efficacy(CE/3) | Num 8 | 1 = subject eliminated from the ATP population for efficacy for elimination type |  |  |
| ELI_F3CM | Elim Efficacy(CM/3) | Num 8 | 1 = subject eliminated from the ATP population for efficacy for elimination type |  |  |
| ELI_F3HS | Elim Efficacy(HS/3) | Num 8 |  |  |  |
| ELI_F3HV | Elim Efficacy(HV/3) | Num 8 | 1 = subject eliminated from the ATP population for efficacy for elimination type |  |  |
| ELI_F3IE | Elim Efficacy(IE/3) | Num 8 | 1 = subject eliminated from the ATP population for efficacy for elimination type |  |  |
| ELI_F3IM | Elim Efficacy(IM/3) | Num 8 | 1 = subject eliminated from the ATP population for efficacy for elimination type |  |  |
| ELI_F3PT | Elim Efficacy(PT/3) | Num 8 | 1 = subject eliminated from the ATP population for efficacy for elimination type |  |  |
| ELI_F3RI | Elim Efficacy(RI/3) | Num 8 | 1 = subject eliminated from the ATP population for efficacy for elimination type |  |  |
| ELI_F3SM | Elim Efficacy(SM/3) | Num 8 | 1 = subject eliminated from the ATP population for efficacy for elimination type |  |  |
| ELI_F4CE | Elim Efficacy(CE/4) | Num 8 | 1 = subject eliminated from the ITT population for elimination type |  |  |
| ELI_F4CM | Elim Efficacy(CM/4) | Num 8 |  |  |  |
| ELI_F4HS | Elim Efficacy(HS/4) | Num 8 | 1 = subject eliminated from the ITT population for elimination type |  |  |
| ELI_F4HV | Elim Efficacy(HV/4) | Num 8 |  |  |  |
| ELI_F4IE | Elim Efficacy(IE/4) | Num 8 | 1 = subject eliminated from the ITT population for elimination type |  |  |
| ELI_F4IM | Elim Efficacy(IM/4) | Num 8 |  |  |  |
| ELI_F4PT | Elim Efficacy(PT/4) | Num 8 |  |  |  |
| ELI_F4RI | Elim Efficacy(RI/4) | Num 8 |  |  |  |
| ELI_F4SM | Elim Efficacy(SM/4) | Num 8 |  |  |  |
| ELI_F5CE | Elim Efficacy(CE/5) | Num 8 | 1 = subject eliminated from the ATP population for efficacy for elimination type |  |  |
| ELI_F5CM | Elim Efficacy(CM/5) | Num 8 | 1 = subject eliminated from the ATP population for efficacy for elimination type |  |  |
| ELI_F5HS | Elim Efficacy(HS/5) | Num 8 | 1 = subject eliminated from the ATP population for efficacy for elimination type |  |  |
| ELI_F5HV | Elim Efficacy(HV/5) | Num 8 | 1 = subject eliminated from the ATP population for efficacy for elimination type |  |  |
| ELI_F5IE | Elim Efficacy(IE/5) | Num 8 | 1 = subject eliminated from the ATP population for efficacy for elimination type |  |  |
| ELI_F5IM | Elim Efficacy(IM/5) | Num 8 | 1 = subject eliminated from the ATP population for efficacy for elimination type |  |  |
| ELI_F5PT | Elim Efficacy(PT/5) | Num 8 | 1 = subject eliminated from the ATP population for efficacy for elimination type |  |  |
| ELI_F5RI | Elim Efficacy(RI/5) | Num 8 | 1 = subject eliminated from the ATP population for efficacy for elimination type |  |  |
| ELI_F5SM | Elim Efficacy(SM/5) | Num 8 | 1 = subject eliminated from the ATP population for efficacy for elimination type |  |  |
| ENDATB | End date of FU period for cohort ATB | Num 8 |  |  | End of ATP [M20-dec13] |
| ENDATB_SE | End date of FU period for cohort ATB | Num 8 |  |  | End of ATP [M20-M32] |
| ENDATP | End date of FU period for cohort ATP | Num 8 |  |  | End of ATP [M2.5-dec13] |
| ENDATP_SE | End date of FU period for cohort ATP | Num 8 |  |  | End of ATP [M2.5-M32] |
| ENDGAP | Extension consent date | Num 8 |  |  | End of potential data gap between M32 and start extension period (until dec 13 for all subjects) |
| ENDITB | End date of FU period for cohort ITB | Num 8 |  |  | End of ITT [M20-dec13] |
| ENDITB_SE | End date of FU period for cohort ITB | Num 8 |  |  | End of ITT [M20-M32] |
| ENDITT | End date of FU period for cohort ITT | Num 8 |  |  | End of ITT [M0-dec13] |
| ENDITT_SE | End date of FU period for cohort ITT | Num 8 |  |  | End of ITT [M0-M32] |
| F2200CAD | F2200CAD | Char 1 | N = No |  | See pid.f2200cad |
|  |  |  | Y = Yes |  |  |
| F2200OLD | F2200OLD | Char 1 | N = No |  | See pid.f2200old |
|  |  |  | Y = Yes |  |  |
| F6000OLD | F6000OLD | Char 1 | N = No |  | See pid.f6000old |
|  |  |  | Y = Yes |  |  |
| GROUP_NB | Group number | Num 8 | 1 = R3R |  | See pid.group_nb |
|  |  |  | 2 = R3C |  |  |
|  |  |  | 3 = C3C |  |  |
| IN_EXTENSION | IN_EXTENSION | Num 8 |  |  | Consented to extension phase (M32-Dec13) |
| PID | Subject Identification | Num 8 |  |  | From *.pid field on the first page of the CRF |
| RND_ID | Treatment number | Num 8 |  |  | See pid.rnd_id |
| SCHEDULE | Schedule (clinical effective) | Num 8 |  |  | See pid.schedule |
| SITE | Site | Char 9 | Agogo |  | See pid.site |
|  |  |  | Bagamoyo |  |  |
|  |  |  | Kilifi |  |  |
|  |  |  | Kintampo |  |  |
|  |  |  | Kombewa |  |  |
|  |  |  | Korogwe |  |  |
|  |  |  | Lambarene |  |  |
|  |  |  | Lilongwe |  |  |
|  |  |  | Manhica |  |  |
|  |  |  | Nanoro |  |  |
|  |  |  | Siaya |  |  |
| STRTATB | Start date of FU period for cohort ATB | Num 8 |  |  | start of ATP [M20-dec13] |
| STRTATP | Start date of FU period for cohort ATP | Num 8 |  |  | start of ATP [M2.5-dec13] |
| STRTGAP | Visit 34 date (start of potential gap) | Num 8 |  |  | Start of potential data gap between M32 and start extension period (until dec 13 for all subjects) |
| STRTITB | Start date of FU period for cohort ITB | Num 8 |  |  | start of ITT [M20-dec13] |
| STRTITT | Start date of FU period for cohort ITT | Num 8 |  |  | start of ITT [M0-dec13] |
| TREATMNT | Treatment | Char 200 | Control |  | See pid.treatmnt |
|  |  |  | RTS,S/AS01E primary schedule with booster |  |  |
|  |  |  | RTS,S/AS01E primary schedule without booster |  |  |

Study 110021 – orgalladmittee

| **Variable** | **Description** | **Type** | **Codes** | **Crf Page** | **Comment** |
| --- | --- | --- | --- | --- | --- |
| ACIDOSIS | Acidosis | Num 8 |  |  | Derived: 1 if base excess <= -10 and be not missing. 0 otherwise |
| AGE | Age | Num 8 |  |  | Derived: difference in months between the subject’s date of birth and the admission date |
| ANEMIA | Anemia | Num 8 |  |  | Derived: 1 if hemoglobin value is less than 5 and not missing. 0 Otherwise |
| ANEMIA_FP | Anemia during entire admission | Num 8 |  |  |  |
| BCS | Blantyre Coma Scale | Num 8 |  |  | Derived: per protocol |
| BC_FP | Blood culture positive entire admission | Num 8 | 0 = No |  |  |
|  |  |  | 1 = Yes |  |  |
| BLTRANS | Blood transfusion | Num 8 |  |  |  |
| BL_TRANS | Blood transfusion | Char 2 | N = No |  |  |
|  |  |  | Y = Yes |  |  |
| COMORB | Any comorbidity? | Num 8 | 0 = No |  | Derived: 1 if the sum of the derived variables dev_bc, dev_csf, dev_cxr and dev_ge is greater than 0. 0 otherwise. |
|  |  |  | 1 = Yes |  |  |
| CO_BC | Comorbidity positive blood culture | Num 8 | 0 = No |  | Derived: 1 if the subject has positive blood culture comorbidity during an admission. 0 otherwise |
|  |  |  | 1 = Yes |  |  |
| CO_GE | Comorbidity gastro enteritis | Num 8 | 0 = No |  | Derived: 1 if the subject has gastro enteritis comorbidity during an admission. 0 otherwise |
|  |  |  | 1 = Yes |  |  |
| CO_MEN | Comorbidity meningitis | Num 8 | 0 = No |  | Derived: 1 if the subject has meningitis comorbidity during an admission. 0 otherwise |
|  |  |  | 1 = Yes |  |  |
| CO_PNEU2 | CXR consol/pleural effus/oth infiltrates | Num 8 | 0 = No |  | Derived: CXR consolidation or pleural effusion or other infiltrates comorbidity. Derived: |
|  |  |  | 1 = Yes |  |  |
| CO_PNEUM | Comorbidity pneumonia | Num 8 | 0 = No |  | Derived: 1 if the subject has pneumonia comorbidity during an admission. 0 otherwise |
|  |  |  | 1 = Yes |  |  |
| CSF_YN | CSF done | Char 2 | Y = Yes |  |  |
| CXR | CXR indicated? | Num 8 | 0 = No |  | Derived: |
|  |  |  | 1 = Yes |  |  |
| DATE | Admission Date | Num 8 |  | crf1:184, 196 | labo.adm_dt  Date of admission, taken from dataset efficacy. |
|  |  |  |  | crf1:184, 196 | signs.adm_dt  Date of admission, taken from dataset efficacy. |
|  |  |  |  | crf1:184 | symp_his.adm_dt  Date of admission, taken from dataset efficacy. |
| DEVIATED | Any deviation? | Num 8 | 0 = No |  | Derived: 1 if dev_csf=1 OR dev_cxr=1 OR dev_bc=1 OR dev_ge=1. 0 otherwise |
|  |  |  | 1 = Yes |  |  |
| DEV_BC | Deviation blood culture | Num 8 | 0 = No |  | Derived: 1 if co_bc is missing. 0 otherwise |
|  |  |  | 1 = Yes |  |  |
| DEV_CSF | Deviation csf | Num 8 | 0 = No |  | Derived: 1 if lp=1 (LP indicated) and co_men is missing. 0 otherwise |
|  |  |  | 1 = Yes |  |  |
| DEV_CXR | Deviation cxr | Num 8 | 0 = No |  | Derived: 1 if cxr=1 (CXR indicated) and co_pneum is missing. 0 otherwise. |
|  |  |  | 1 = Yes |  |  |
| DEV_GE | Deviation gastro enteritis | Num 8 | 0 = No |  | Derived: |
| FATAL10_ | Fatal malaria primary case definition | Num 8 |  |  | See Analysis plan |
| FATAL21_ | Fatal malaria secondary case def 1 | Num 8 |  |  |  |
| FATAL22_ | Fatal malaria secondary case def 2 | Num 8 |  |  |  |
| FATAL23_ | Fatal malaria secondary case def 3 | Num 8 |  |  |  |
| FINALDEN | Parasite density | Num 8 |  |  | See wslide.a_pf_den  Maximum value of a_pf_den for a pid/adm_dt |
| HIV_POS | HIV positive | Num 8 | 0 = No |  | adhiv.hiv_pos |
|  |  |  | 1 = Yes |  |  |
| HOSPIT | Medical hospitalization case def 1 | Num 8 |  |  | See Analysis plan |
| HYPOGLYC | Hypoglycemia | Num 8 |  |  | Derived: 1 if glucose < 2.2 and glu not missing. 0 otherwise. |
| LACTATE | Lactate | Num 8 |  |  | Derived: 1 if lactate >= 5. 0 otherwise |
| LC_NEURFIN | LC_NEURFIN | Char 47 |  |  |  |
| LC_NEURFU | Neurological outcome follow-ups | Char 47 |  |  |  |
| LC_NEUR_OUT | Neurological outcome | Char 47 |  |  |  |
| LP | LP indicated? | Num 8 | 0 = No |  | Derived: |
|  |  |  | 1 = Yes |  |  |
| MAL_HOS1 | Malaria hospitalization case def 1 | Num 8 |  |  | See Analysis plan |
| MAL_HOS2 | Malaria hospitalization case def 2 | Num 8 |  |  |  |
| MARKER | Any marker? | Num 8 | 0 = No |  | Derived: 1 if at least one of the 7 marker variables (anemia, Prostration, Resp_distress, Seizures, Hypoglyc, Acidosis, Lactate) has value 1 or if bcs is less than or equal to 2 and not missing. 0 if none of the 7 marker variables has value 1 and bcs is greater than 2 or missing. |
|  |  |  | 1 = Yes |  |  |
| NEURFIN | Final Neurological Outcome | Char 2 | 1 |  |  |
|  |  |  | 3 |  |  |
|  |  |  | 4 |  |  |
|  |  |  | 6 |  |  |
| NEURFU | Neurological outcome follow-up | Char 2 |  |  |  |
|  |  |  | 1 |  |  |
|  |  |  | 3 |  |  |
|  |  |  | 4 |  |  |
|  |  |  | 6 |  |  |
| NEUR_OUT | Neurological outcome | Char 2 | 1 |  |  |
|  |  |  | 2 |  |  |
|  |  |  | 3 |  |  |
|  |  |  | 4 |  |  |
|  |  |  | 6 |  |  |
| OUTCOME | Outcome | Char 10 | Abscontion |  |  |
|  |  |  | Death |  |  |
|  |  |  | Discharge |  |  |
| PID | Subject Identification | Num 8 |  |  | From *.pid field on the first page of the CRF |
| PI_MAL | PI s Diagnosis of malaria | Char 2 | N = No |  | See Analysis plan |
|  |  |  | Y = Yes |  |  |
| PNEUM10_ | Primary case definition pneumonia | Num 8 |  |  | See Analysis plan |
| PNEUM21_ | Secondary case definition 1 pneumonia | Num 8 |  |  |  |
| PNEUM22_ | Secondary case definition 2 pneumonia | Num 8 |  |  |  |
| PNEUM23_ | Secondary case definition 2 pneumonia | Num 8 |  |  |  |
| POSS_MEN | Possible meningitis | Num 8 | 0 = No |  |  |
|  |  |  | 1 = Yes |  |  |
| PROSTRAT | Prostration | Num 8 |  |  | Derived: 1 if signs.prostrat=”Y”. 0 otherwise |
| REQ3RDS | Were three readings required? | Num 8 |  |  | See wslide.req3rds |
| RESPDIST | Respiratory distress | Num 8 |  |  | Derived: 1 if signs.lowchest=Y or signs.breath=Y. 0 otherwise |
| S1_DENS | Slide 1 density | Char 7 |  |  | See wslide.s1_dens  Raw parasite density read 1 associated with the selected a_pf_den from w_slide |
| S2_DENS | Slide 2 density | Char 7 |  |  | See wslide.s2_dens  Raw parasite density read 2 associated with the selected a_pf_den from w_slide |
| S3_DENS | Slide 3 density | Char 7 |  |  | See wslide.s3_dens  Raw parasite density read 3 associated with the selected a_pf_den from w_slide |
| SALM_SEP | Sepsis case def 2 Salmonella sepsis | Num 8 |  |  | See Analysis plan |
| SEIZURES | Seizures | Num 8 |  |  | Derived: |
| SEPSIS | Sepsis case definition 1 | Num 8 |  |  | See Analysis plan |
| SEV10_ | Severe malaria primary case definition | Num 8 |  |  | See Analysis plan |
| SEV21_ | Severe malaria secondary case def 1 | Num 8 |  |  |  |
| SEV22_ | Severe malaria secondary case def 2 | Num 8 |  |  |  |
| SEV23_ | Severe malaria secondary case def 3 | Num 8 |  |  |  |
| SEV_AN1_ | Incident severe anaemia case def 1 | Num 8 |  |  | See Analysis plan |
| SEV_AN2_ | Incident severe anaemia case def 2 | Num 8 |  |  |  |
| SEV_AN3_ | Incident severe anaemia case def 3 | Num 8 |  |  |  |
| RES_CODED | Culture result | Char 20 | 1 Pneumocossu  2 Haemofilus influenza  3 Meningococcus  4 Salmonelle  5 Other pathogenic organism  6 Contaminant  7 No organism  8 No result (result missing confirmed)  MC missing confirmed (test not done) |  |  |

Study 110021 - pid

| **Variable** | **Description** | **Type** | **Codes** | **Crf Page** | **Comment** |
| --- | --- | --- | --- | --- | --- |
| AGEC | Age Group (N) | Num 8 | 1 = [5-17] months |  | Derived: if 5<=age<= 17 months then AGEC=1; if 6<=age<= 12 weeks then AGEC=2 |
|  |  |  | 2 = [6-12] weeks |  |  |
| AGE_LABEL | Age Group | Char 13 |  |  | Derived: if 5<=age<= 17 months then AGE_LABEL=”[5-17] months”; if 6<=age<= 12 weeks then AGE_LABEL=”[6-12] weeks” |
| BLOCK_NB | Randomization Block number | Num 8 |  |  | Derived: From randomisation system. For internal use only. |
| CENTER | Center number | Char 10 |  |  | demog.center |
| CTY_COD | Country code | Char 3 | BF = Burkina Faso |  | From data management. Code of the country where the center (center) is located. |
|  |  |  | GA = Gabon |  |  |
|  |  |  | GH = Ghana |  |  |
|  |  |  | KE = Kenya |  |  |
|  |  |  | MW = Malawi |  |  |
|  |  |  | MZ = Mozambique |  |  |
|  |  |  | TZ = Tanzania |  |  |
| CTY_NAM | Country name | Char 30 | Burkina Faso |  | From data management. Name of the country where the center (center) is located. |
|  |  |  | Gabon |  |  |
|  |  |  | Ghana |  |  |
|  |  |  | Kenya |  |  |
|  |  |  | Malawi |  |  |
|  |  |  | Mozambique |  |  |
|  |  |  | Tanzania |  |  |
| ELIG_CE | Eligibility(CE) | Char 1 | E = subject eliminated from the ATP cohort for safety and the ATP cohort for immunogenicity for elimination type |  | See Elim codes overview |
|  |  |  | I = subject eliminated from the ATP cohort for immunogenicity but not from the ATP cohort for safety for elimination type |  |  |
| ELIG_CM | Eligibility(CM) | Char 1 | E = subject eliminated from the ATP cohort for safety and the ATP cohort for immunogenicity for elimination type |  |  |
|  |  |  | I = subject eliminated from the ATP cohort for immunogenicity but not from the ATP cohort for safety for elimination type |  |  |
| ELIG_HS | Eligibility(HS) | Char 1 | I = subject eliminated from the ATP cohort for immunogenicity but not from the ATP cohort for safety for elimination type |  |  |
| ELIG_HV | Eligibility(HV) | Char 1 | E = subject eliminated from the ATP cohort for safety and the ATP cohort for immunogenicity for elimination type |  |  |
|  |  |  | I = subject eliminated from the ATP cohort for immunogenicity but not from the ATP cohort for safety for elimination type |  |  |
| ELIG_IE | Eligibility(IE) | Char 1 | E = subject eliminated from the ATP cohort for safety and the ATP cohort for immunogenicity for elimination type |  |  |
|  |  |  | I = subject eliminated from the ATP cohort for immunogenicity but not from the ATP cohort for safety for elimination type |  |  |
| ELIG_IM | Eligibility(IM) | Char 1 | E = subject eliminated from the ATP cohort for safety and the ATP cohort for immunogenicity for elimination type |  |  |
|  |  |  | I = subject eliminated from the ATP cohort for immunogenicity but not from the ATP cohort for safety for elimination type |  |  |
| ELIG_PT | Eligibility(PT) | Char 1 | E = subject eliminated from the ATP cohort for safety and the ATP cohort for immunogenicity for elimination type |  |  |
|  |  |  | I = subject eliminated from the ATP cohort for immunogenicity but not from the ATP cohort for safety for elimination type |  |  |
| ELIG_RI | Eligibility(RI) | Char 1 | E = subject eliminated from the ATP cohort for safety and the ATP cohort for immunogenicity for elimination type |  |  |
|  |  |  | I = subject eliminated from the ATP cohort for immunogenicity but not from the ATP cohort for safety for elimination type |  |  |
| ELIG_SM | Eligibility(SM) | Char 1 | E = subject eliminated from the ATP cohort for safety and the ATP cohort for immunogenicity for elimination type |  |  |
|  |  |  | I = subject eliminated from the ATP cohort for immunogenicity but not from the ATP cohort for safety for elimination type |  |  |
| ELIM_RCE | Elim reacto(CE) | Num 8 | 1 = subject eliminated from the ATP cohort for safety for elimination type |  |  |
| ELIM_RCM | Elim reacto(CM) | Num 8 | 1 = subject eliminated from the ATP cohort for safety for elimination type |  |  |
| ELIM_RHS | Elim reacto(HS) | Num 8 |  |  |  |
| ELIM_RHV | Elim reacto(HV) | Num 8 | 1 = subject eliminated from the ATP cohort for safety for elimination type |  |  |
| ELIM_RIE | Elim reacto(IE) | Num 8 | 1 = subject eliminated from the ATP cohort for safety for elimination type |  |  |
| ELIM_RIM | Elim reacto(IM) | Num 8 | 1 = subject eliminated from the ATP cohort for safety for elimination type |  |  |
| ELIM_RPT | Elim reacto(PT) | Num 8 | 1 = subject eliminated from the ATP cohort for safety for elimination type |  |  |
| ELIM_RRI | Elim reacto(RI) | Num 8 | 1 = subject eliminated from the ATP cohort for safety for elimination type |  |  |
| ELIM_RSM | Elim reacto(SM) | Num 8 | 1 = subject eliminated from the ATP cohort for safety for elimination type |  |  |
| ELIM_SCE | Elim sero(CE) | Num 8 | 1 = subject eliminated from the ATP cohort for immunogenicity for elimination type |  |  |
| ELIM_SCM | Elim sero(CM) | Num 8 | 1 = subject eliminated from the ATP cohort for immunogenicity for elimination type |  |  |
| ELIM_SHS | Elim sero(HS) | Num 8 | 1 = subject eliminated from the ATP cohort for immunogenicity for elimination type |  |  |
| ELIM_SHV | Elim sero(HV) | Num 8 | 1 = subject eliminated from the ATP cohort for immunogenicity for elimination type |  |  |
| ELIM_SIE | Elim sero(IE) | Num 8 | 1 = subject eliminated from the ATP cohort for immunogenicity for elimination type |  |  |
| ELIM_SIM | Elim sero(IM) | Num 8 | 1 = subject eliminated from the ATP cohort for immunogenicity for elimination type |  |  |
| ELIM_SPT | Elim sero(PT) | Num 8 | 1 = subject eliminated from the ATP cohort for immunogenicity for elimination type |  |  |
| ELIM_SRI | Elim sero(RI) | Num 8 | 1 = subject eliminated from the ATP cohort for immunogenicity for elimination type |  |  |
| ELIM_SSM | Elim sero(SM) | Num 8 | 1 = subject eliminated from the ATP cohort for immunogenicity for elimination type |  |  |
| ELIM_ST | Elim Stat | Num 8 |  |  |  |
| ELIM_V | Elim Vaccin | Num 8 |  |  |  |
| ELI_F3CE | Elim Efficacy(CE/3) | Num 8 | 1 = subject eliminated from the ATP population for efficacy for elimination type |  |  |
| ELI_F3CM | Elim Efficacy(CM/3) | Num 8 | 1 = subject eliminated from the ATP population for efficacy for elimination type |  |  |
| ELI_F3HS | Elim Efficacy(HS/3) | Num 8 |  |  |  |
| ELI_F3HV | Elim Efficacy(HV/3) | Num 8 | 1 = subject eliminated from the ATP population for efficacy for elimination type |  |  |
| ELI_F3IE | Elim Efficacy(IE/3) | Num 8 | 1 = subject eliminated from the ATP population for efficacy for elimination type |  |  |
| ELI_F3IM | Elim Efficacy(IM/3) | Num 8 | 1 = subject eliminated from the ATP population for efficacy for elimination type |  |  |
| ELI_F3PT | Elim Efficacy(PT/3) | Num 8 | 1 = subject eliminated from the ATP population for efficacy for elimination type |  |  |
| ELI_F3RI | Elim Efficacy(RI/3) | Num 8 | 1 = subject eliminated from the ATP population for efficacy for elimination type |  |  |
| ELI_F3SM | Elim Efficacy(SM/3) | Num 8 | 1 = subject eliminated from the ATP population for efficacy for elimination type |  |  |
| ELI_F4CE | Elim Efficacy(CE/4) | Num 8 | 1 = subject eliminated from the ITT population for elimination type |  |  |
| ELI_F4CM | Elim Efficacy(CM/4) | Num 8 |  |  |  |
| ELI_F4HS | Elim Efficacy(HS/4) | Num 8 | 1 = subject eliminated from the ITT population for elimination type |  |  |
| ELI_F4HV | Elim Efficacy(HV/4) | Num 8 |  |  |  |
| ELI_F4IE | Elim Efficacy(IE/4) | Num 8 | 1 = subject eliminated from the ITT population for elimination type |  |  |
| ELI_F4IM | Elim Efficacy(IM/4) | Num 8 |  |  |  |
| ELI_F4PT | Elim Efficacy(PT/4) | Num 8 |  |  |  |
| ELI_F4RI | Elim Efficacy(RI/4) | Num 8 |  |  |  |
| ELI_F4SM | Elim Efficacy(SM/4) | Num 8 |  |  |  |
| ELI_F5CE | Elim Efficacy(CE/5) | Num 8 | 1 = subject eliminated from the ATP population for efficacy for elimination type |  |  |
| ELI_F5CM | Elim Efficacy(CM/5) | Num 8 | 1 = subject eliminated from the ATP population for efficacy for elimination type |  |  |
| ELI_F5HS | Elim Efficacy(HS/5) | Num 8 | 1 = subject eliminated from the ATP population for efficacy for elimination type |  |  |
| ELI_F5HV | Elim Efficacy(HV/5) | Num 8 | 1 = subject eliminated from the ATP population for efficacy for elimination type |  |  |
| ELI_F5IE | Elim Efficacy(IE/5) | Num 8 | 1 = subject eliminated from the ATP population for efficacy for elimination type |  |  |
| ELI_F5IM | Elim Efficacy(IM/5) | Num 8 | 1 = subject eliminated from the ATP population for efficacy for elimination type |  |  |
| ELI_F5PT | Elim Efficacy(PT/5) | Num 8 | 1 = subject eliminated from the ATP population for efficacy for elimination type |  |  |
| ELI_F5RI | Elim Efficacy(RI/5) | Num 8 | 1 = subject eliminated from the ATP population for efficacy for elimination type |  |  |
| ELI_F5SM | Elim Efficacy(SM/5) | Num 8 | 1 = subject eliminated from the ATP population for efficacy for elimination type |  |  |
| F2200CAD | F2200CAD | Char 1 | N = No |  |  |
|  |  |  | Y = Yes |  |  |
| F2200OLD | F2200OLD | Char 1 | N = No |  |  |
|  |  |  | Y = Yes |  |  |
| F6000OLD | F6000OLD | Char 1 | N = No |  |  |
|  |  |  | Y = Yes |  |  |
| GROUP_NB | Group number | Num 8 | 1 = R3R |  |  |
|  |  |  | 2 = R3C |  |  |
|  |  |  | 3 = C3C |  |  |
| HIV | HIV | Num 8 |  |  | Derived- If pid is confirmed HIV positive |
| PID | Subject Identification | Num 8 |  |  | From *.pid field on the first page of the CRF |
| PRETERM | PRETERM | Num 8 |  |  |  |
| RND_ID | Effective randomization number | Num 8 |  |  | Derived: From randomisation system. For internal use only. |
| SCHEDULE | Schedule | Num 8 |  |  | Derived: Clinical schedule used for statistical analysis. |
| SITE | Site | Char 9 | Agogo |  |  |
|  |  |  | Bagamoyo |  |  |
|  |  |  | Kilifi |  |  |
|  |  |  | Kintampo |  |  |
|  |  |  | Kombewa |  |  |
|  |  |  | Korogwe |  |  |
|  |  |  | Lambarene |  |  |
|  |  |  | Lilongwe |  |  |
|  |  |  | Manhica |  |  |
|  |  |  | Nanoro |  |  |
|  |  |  | Siaya |  |  |
| SITEC2 | Site (N) | Num 8 | 1 = Agogo |  | Derived: |
|  |  |  | 10 = Nanoro |  |  |
|  |  |  | 11 = Siaya |  |  |
|  |  |  | 2 = Bagamoyo |  |  |
|  |  |  | 3 = Kilifi |  |  |
|  |  |  | 4 = Kintampo |  |  |
|  |  |  | 5 = Kombewa |  |  |
|  |  |  | 6 = Korogwe |  |  |
|  |  |  | 7 = Lambarene |  |  |
|  |  |  | 8 = Lilongwe |  |  |
|  |  |  | 9 = Manhica |  |  |
| TREATMNT | Treatment | Char 200 | Control |  | From protocol. |
|  |  |  | RTS,S/AS01E primary schedule with booster |  |  |
|  |  |  | RTS,S/AS01E primary schedule without booster |  |  |
| VERYLOW_WAZ | VERYLOW_WAZ | Num 8 |  |  |  |
| WAZCAT | WAZ at Month 20 category (N) | Num 8 | 0 = <=-3 |  | Derived: if WAZ at month 20 <=-3 then WAZCAT=0; if -3<WAZ at month 20 <=-2 then WAZCAT=1; if WAZ at month 20>=-2 then WAZCAT=2; else WAZCAT=9 |
|  |  |  | 1 = ]-3,-2] |  |  |
|  |  |  | 2 = >-2 |  |  |
|  |  |  | 9 = Missing |  |  |
| WAZCAT_BSL | WAZ at baseline category (N) | Num 8 | 0 = <=-3 |  | Derived: if WAZ at baseline <=-3 then WAZCAT=0; if -3<WAZ at baseline <=-2 then WAZCAT=1; if WAZ at baseline>=-2 then WAZCAT=2; else WAZCAT=9 |
|  |  |  | 1 = ]-3,-2] |  |  |
|  |  |  | 2 = >-2 |  |  |
|  |  |  | 9 = Missing |  |  |

Study 110021 - serocod

| **Variable** | **Description** | **Type** | **Codes** | **Crf Page** | **Comment** |
| --- | --- | --- | --- | --- | --- |
| ACTIVITY | Activity | Num 8 | 10 = SCREENING (VISIT1) OR VISIT1 |  | From *.activity field on top of first page of each CRF segment |
|  |  |  | 20 = PRE (VISIT2) OR VISIT2 |  |  |
|  |  |  | 220 = PIII(M20) (VISIT22) OR VISIT22 |  |  |
|  |  |  | 230 = PIV(M21) (VISIT23) OR VISIT23 |  |  |
|  |  |  | 340 = PIV(M32) (VISIT34) OR VISIT34 |  |  |
|  |  |  | 360 = PIV(M44) (VISIT36) OR VISIT36 |  |  |
|  |  |  | 380 = PIV(M56) (VISIT38) OR VISIT38 |  |  |
|  |  |  | 50 = PIII(M3) (VISIT5) OR VISIT5 |  |  |
| ACTREF_1 | Reference activity 1 | Num 8 | 10 = VISIT1 |  | From analysis plan. Reference activity for fold increase (see serocod.r) |
| ACTREF_2 | Reference activity 2 | Num 8 | 10 = VISIT1 |  | From analysis plan. Reference activity for second fold increase (see serocod.r_2) |
| ACT_SSBV | Pre-vaccination reference activity | Num 8 | . |  | From analysis plan. Reference activity for pre-vaccination status (see serocod.ssbv) |
|  |  |  | 10 = VISIT1 |  |  |
| COMPO | Component | Char 20 | Code: |  | Derived: w_labo.testdesc |
|  |  |  | HBV.S AB |  |  |
|  |  |  | HIV AB |  |  |
|  |  |  | HIV PCR |  |  |
|  |  |  | P FALCI.CSP.R32LR IG |  |  |
|  |  |  | POLIO 1 AB |  |  |
|  |  |  | POLIO 2 AB |  |  |
|  |  |  | POLIO 3 AB |  |  |
| COMPONEN | Component | Char 20 |  |  | Derived: w_labo.newtest |
| COMPO_UN | Component (unit) | Char 43 | Code: ( ) |  | Derived: concatenation of variables compo and unit |
|  |  |  | HBV.S AB (MIU/ML) |  |  |
|  |  |  | HIV AB ( ) |  |  |
|  |  |  | HIV PCR ( ) |  |  |
|  |  |  | P FALCI.CSP.R32LR IG (ELU/ML) |  |  |
|  |  |  | POLIO 1 AB (ED50) |  |  |
|  |  |  | POLIO 2 AB (ED50) |  |  |
|  |  |  | POLIO 3 AB (ED50) |  |  |
| CUT_OFF | Cut-Off | Num 8 | . |  | From laboratory specifications. Seropositivity threshold (seropositive if (num_res)>=(cut_off)) |
|  |  |  | 1 |  |  |
|  |  |  | 10 |  |  |
|  |  |  | 8 |  |  |
| EVENTTYP | Sampling type | Char 4 | SER = SERUM |  | Derived: w_labo.samptype |
| GROUP_NB | Group number | Num 8 | 1 = R3R |  | See pid.group_nb |
|  |  |  | 2 = R3C |  |  |
|  |  |  | 3 = C3C |  |  |
| LAB_CODE | Laboratory code | Char 2 |  |  | From laboratory results. INTERNAL USE ONLY |
| LOG_VAL | Log of the concentration/titre | Num 8 |  |  | Derived: The log10 of NUM_RES |
| METHOD | Measurement method used | Char 20 |  |  | From laboratory specifications. _TO_CHECK_ |
| N | Test result available and valid? | Num 8 | 0 = the serological result of the test is not available for the subject |  | Derived: set to 1 if (num_res) contains a valid numerical value, else 0. |
|  |  |  | 1 = the serological result of the test is available for the subject |  |  |
| NEWTEST | Test Code (Clinical) | Char 5 | 452 = P FALCI.CSP.R32LR IG - ELISA - ELU/ML |  | Derived: alphanumerical formats of serotest |
|  |  |  | 871 = HBV.S AB - ELISA - MIU/ML |  |  |
|  |  |  | 907 = POLIO 1 AB - NEUTRA - ED50 |  |  |
|  |  |  | 908 = POLIO 2 AB - NEUTRA - ED50 |  |  |
|  |  |  | 909 = POLIO 3 AB - NEUTRA - ED50 |  |  |
|  |  |  | HIV = HIV AB |  |  |
|  |  |  | HIV_P = HIV PCR |  |  |
| NUM_RES | Serological results | Num 8 |  |  | Derived: serological results as a number : (rawres) converted to a number. See note 1 for more details. |
| P | Subject is seroprotected ? | Num 8 |  |  | Derived: missing if no valid numerical result, 1 if (num_res) >= (PROTECT), 0 if (num_res) < (PROTECT). ALL VALUES MISSING |
| PID | Subject Identification | Num 8 |  |  | From *.pid field on the first page of the CRF |
| PROTECT | Protection level | Num 8 | . |  | From laboratory specifications. Seroprotection threshold (seroprotected if (num_res)>=(protect)) ALL VALUES MISSING |
| R | Comparison ratio 1 | Num 8 |  |  | Derived: comparison ratio between two time points for the current subject (pid) and test (serotest). It is the ratio of current (num_res) over (num_res) at activity specified in (actref_1). |
| RAWRES | Raw results | Char 10 | BS NA = BS Not Applicable |  | From laboratory results. Results of serology test |
|  |  |  | BS ND = BS Not Done |  |  |
|  |  |  | IR = Invalid Result |  |  |
|  |  |  | N = Negative |  |  |
|  |  |  | ND = Not Done |  |  |
|  |  |  | P = Positive |  |  |
|  |  |  | QNS = Quantity Not Sufficient |  |  |
|  |  |  | SAMP NAV = Sample not available |  |  |
|  |  |  | VIS ND = Visit Not Done |  |  |
| RESVAL | Result validity | Char 3 | N = No |  | Derived: Result validity |
|  |  |  | NA = Not applicable |  |  |
|  |  |  | Y = Yes |  |  |
| R_2 | Comparison ratio 2 | Num 8 |  |  | Derived: comparison ratio between two time points for the current subject (pid) and test (serotest). It is the ratio of current (num_res) over (num_res) at activity specified in (actref_2). |
| S | Subject is seropositive ? | Num 8 | 0 = the subject is seronegative |  | Derived: missing if no valid numerical result, 1 if (num_res)>= (CUT_OFF), 0 if (num_res) < (CUT_OFF). |
|  |  |  | 1 = the subject is seropositive |  |  |
| SAMPSTAT | Sample status | Char 8 | NR = Result not available |  | Derived: Sample status |
|  |  |  | RES = Result available |  |  |
|  |  |  | SAMP MC = Sample missing |  |  |
|  |  |  | SAMP NA = Sample not applicable |  |  |
|  |  |  | SAMP NAV = Sample not available |  |  |
|  |  |  | SAMP ND = Sample not done |  |  |
|  |  |  | VIS ND = Visit not done |  |  |
|  |  |  | VIS UNK = Visit unknown |  |  |
| SAMPTAKE | Sampling [Y/N] | Char 2 | MC = MISSING CONFIRMED | crf1:3, 28, 66, 93, 94, 108, 126, 127, 141, 142, 143, 147, 148, 149 | labsheet.samptake |
|  |  |  | N = NO |  |  |
|  |  |  | NA = NOT APPLICABLE |  |  |
|  |  |  | Y = YES |  |  |
| SAMPTYPE | Sampling type | Char 3 | BL = IMMUNOGENICITY |  | labsheet.samptype |
|  |  |  | BL1 = BL - IMMUNOGENICITY |  |  |
| SCHEDULE | Schedule (clinical effective) | Num 8 |  |  | See pid.schedule |
| SERONAME | Test description | Char 20 | Anti-CS |  | From laboratory specifications. The name of the anti-antigen tested. |
|  |  |  | Code: |  |  |
|  |  |  | HBV.S AB |  |  |
|  |  |  | HIV AB |  |  |
|  |  |  | HIV PCR |  |  |
|  |  |  | POLIO 1 AB |  |  |
|  |  |  | POLIO 2 AB |  |  |
|  |  |  | POLIO 3 AB |  |  |
| SERON_UN | Concatenation of seroname and unit | Char 43 | Code: ( ) |  | Derived: concatenation of (seroname) and (unit) |
|  |  |  | HBV.S AB (MIU/ML) |  |  |
|  |  |  | HIV AB ( ) |  |  |
|  |  |  | HIV PCR ( ) |  |  |
|  |  |  | P FALCI.CSP.R32LR IG (ELU/ML) |  |  |
|  |  |  | POLIO 1 AB (ED50) |  |  |
|  |  |  | POLIO 2 AB (ED50) |  |  |
|  |  |  | POLIO 3 AB (ED50) |  |  |
| SEROTEST | serotest code | Num 8 | 452 = P FALCI.CSP.R32LR IG - ELISA - ELU/ML |  | From laboratory specifications. Internal code identifying the test. |
|  |  |  | 871 = HBV.S AB - ELISA - MIU/ML |  |  |
|  |  |  | 907 = POLIO 1 AB - NEUTRA - ED50 |  |  |
|  |  |  | 908 = POLIO 2 AB - NEUTRA - ED50 |  |  |
|  |  |  | 909 = POLIO 3 AB - NEUTRA - ED50 |  |  |
| SEROTYPE | Sampling type | Char 3 |  |  | labsheet.samptype |
| SSBV | Seropositivity status | Num 8 | . = missing |  | Derived: value of (s) for the current subject (pid) and test (serotest) but at activity specified in (act_ssbv). |
|  |  |  | 1 = if the subject was seronegative |  |  |
|  |  |  | 2 = if the subject was seropositive |  |  |
| TIMING | Timing of sample | Char 20 |  |  | From protocol |
|  |  |  | PIII(M20) |  |  |
|  |  |  | PIII(M3) |  |  |
|  |  |  | PIV(M21) |  |  |
|  |  |  | PIV(M32) |  |  |
|  |  |  | PIV(M44) |  |  |
|  |  |  | PIV(M56) |  |  |
|  |  |  | PRE |  |  |
|  |  |  | SCREENING |  |  |
| UNIT | Unit used | Char 20 |  |  | From laboratory specifications |
| V_ID | Variant ID | Char 20 |  |  | Derived: from assay dictionary.v_id |

Note 1:

If rawres is ‘NEG’ or ‘-’ or ‘(-)’, num_res= cutt_off/2,

if rawres is ‘POS’ or ‘+’ or ‘(+)’, num_res = cut_off,

if rawres is ‘< value’ and value<=cut_off, num_res=cut_off/2,

if rawres is ‘< value’ and value>cut_off, num_res=value,

if rawres is ‘> value’ and value<cut_off, num_res=cut_off/2,

if rawres is ‘> value’ and value>=cut_off, num_res=value,

if rawres is ‘<= value’ or ‘>= value’ and value<cut_off, num_res=cut_off/2,

if rawres is ‘<= value’ or ‘>= value’ and value>=cut_off, num_res=value,

if rawres is a value < cut_off, num_res = cut_off/2,

if rawres is a value >= cut_off, num_res = rawres,

if rawres is a value >= cut_off, num_res = rawres,

else num_res is left blank.

Study 110021 - sevmal

| **Variable** | **Description** | **Type** | **Codes** | **Crf Page** | **Comment** |
| --- | --- | --- | --- | --- | --- |
| ACIDOSIS | Acidosis | Num 8 |  |  | Derived: 1 if base excess <= -10 and be not missing. 0 otherwise |
| AGE | Age | Num 8 |  |  | Derived: difference in months between the subject’s date of birth and the admission date |
| ANEMIA | Anemia | Num 8 |  |  | Derived: 1 if hemoglobin value is less than 5 and not missing. 0 Otherwise |
| ANEMIA_FP | Anemia during entire admission | Num 8 |  |  |  |
| BCS | Blantyre Coma Scale | Num 8 |  |  | Derived: per protocol |
| BC_FP | Blood culture positive entire admission | Num 8 | 0 = No |  |  |
|  |  |  | 1 = Yes |  |  |
| BLTRANS | Blood transfusion | Num 8 |  |  |  |
| BL_TRANS | Blood transfusion | Char 2 | N = No |  |  |
|  |  |  | Y = Yes |  |  |
| COMORB | Any comorbidity? | Num 8 | 0 = No |  | Derived: 1 if the sum of the derived variables dev_bc, dev_csf, dev_cxr and dev_ge is greater than 0. 0 otherwise. |
|  |  |  | 1 = Yes |  |  |
| CO_BC | Comorbidity positive blood culture | Num 8 | 0 = No |  | Derived: 1 if the subject has positive blood culture comorbidity during an admission. 0 otherwise |
|  |  |  | 1 = Yes |  |  |
| CO_GE | Comorbidity gastro enteritis | Num 8 | 0 = No |  | Derived: 1 if the subject has gastro enteritis comorbidity during an admission. 0 otherwise |
|  |  |  | 1 = Yes |  |  |
| CO_MEN | Comorbidity meningitis | Num 8 | 0 = No |  | Derived: 1 if the subject has meningitis comorbidity during an admission. 0 otherwise |
|  |  |  | 1 = Yes |  |  |
| CO_PNEU2 | CXR consol/pleural effus/oth infiltrates | Num 8 | 0 = No |  | Derived: CXR consolidation or pleural effusion or other infiltrates comorbidity. Derived: |
|  |  |  | 1 = Yes |  |  |
| CO_PNEUM | Comorbidity pneumonia | Num 8 | 0 = No |  | Derived: 1 if the subject has pneumonia comorbidity during an admission. 0 otherwise |
|  |  |  | 1 = Yes |  |  |
| CSF_YN | CSF done | Char 2 | Y = Yes |  |  |
| CXR | CXR indicated? | Num 8 | 0 = No |  | Derived: |
|  |  |  | 1 = Yes |  |  |
| DATE | Admission Date | Num 8 |  | crf1:184, 196 | labo.adm_dt  Date of admission, taken from dataset efficacy. |
|  |  |  |  | crf1:184, 196 | signs.adm_dt  Date of admission, taken from dataset efficacy. |
|  |  |  |  | crf1:184 | symp_his.adm_dt  Date of admission, taken from dataset efficacy. |
| DEVIATED | Any deviation? | Num 8 | 0 = No |  | Derived: 1 if dev_csf=1 OR dev_cxr=1 OR dev_bc=1 OR dev_ge=1. 0 otherwise |
|  |  |  | 1 = Yes |  |  |
| DEV_BC | Deviation blood culture | Num 8 | 0 = No |  | Derived: 1 if co_bc is missing. 0 otherwise |
|  |  |  | 1 = Yes |  |  |
| DEV_CSF | Deviation csf | Num 8 | 0 = No |  | Derived: 1 if lp=1 (LP indicated) and co_men is missing. 0 otherwise |
|  |  |  | 1 = Yes |  |  |
| DEV_CXR | Deviation cxr | Num 8 | 0 = No |  | Derived: 1 if cxr=1 (CXR indicated) and co_pneum is missing. 0 otherwise. |
|  |  |  | 1 = Yes |  |  |
| DEV_GE | Deviation gastro enteritis | Num 8 | 0 = No |  | Derived: |
| FATAL10_ | Fatal malaria primary case definition | Num 8 |  |  | See Analysis plan |
| FATAL21_ | Fatal malaria secondary case def 1 | Num 8 |  |  |  |
| FATAL22_ | Fatal malaria secondary case def 2 | Num 8 |  |  |  |
| FATAL23_ | Fatal malaria secondary case def 3 | Num 8 |  |  |  |
| FINALDEN | Parasite density | Num 8 |  |  | See wslide.a_pf_den  Maximum value of a_pf_den for a pid/adm_dt |
| HIV_POS | HIV positive | Num 8 | 0 = No |  | adhiv.hiv_pos |
|  |  |  | 1 = Yes |  |  |
| HOSPIT | Medical hospitalization case def 1 | Num 8 |  |  | See Analysis plan |
| HYPOGLYC | Hypoglycemia | Num 8 |  |  | Derived: 1 if glucose < 2.2 and glu not missing. 0 otherwise. |
| LACTATE | Lactate | Num 8 |  |  | Derived: 1 if lactate >= 5. 0 otherwise |
| LC_NEURFIN | LC_NEURFIN | Char 47 |  |  |  |
| LC_NEURFU | Neurological outcome follow-ups | Char 47 |  |  |  |
| LC_NEUR_OUT | Neurological outcome | Char 47 |  |  |  |
| LP | LP indicated? | Num 8 | 0 = No |  | Derived: |
|  |  |  | 1 = Yes |  |  |
| MAL_HOS1 | Malaria hospitalization case def 1 | Num 8 |  |  | See Analysis plan |
| MAL_HOS2 | Malaria hospitalization case def 2 | Num 8 |  |  |  |
| MARKER | Any marker? | Num 8 | 0 = No |  | Derived: 1 if at least one of the 7 marker variables (anemia, Prostration, Resp_distress, Seizures, Hypoglyc, Acidosis, Lactate) has value 1 or if bcs is less than or equal to 2 and not missing. 0 if none of the 7 marker variables has value 1 and bcs is greater than 2 or missing. |
|  |  |  | 1 = Yes |  |  |
| NEURFIN | Final Neurological Outcome | Char 2 | 1 |  |  |
|  |  |  | 3 |  |  |
|  |  |  | 4 |  |  |
|  |  |  | 6 |  |  |
| NEURFU | Neurological outcome follow-up | Char 2 |  |  |  |
|  |  |  | 1 |  |  |
|  |  |  | 3 |  |  |
|  |  |  | 4 |  |  |
|  |  |  | 6 |  |  |
| NEUR_OUT | Neurological outcome | Char 2 | 1 |  |  |
|  |  |  | 2 |  |  |
|  |  |  | 3 |  |  |
|  |  |  | 4 |  |  |
|  |  |  | 6 |  |  |
| OUTCOME | Outcome | Char 10 | Abscontion |  |  |
|  |  |  | Death |  |  |
|  |  |  | Discharge |  |  |
| PID | Subject Identification | Num 8 |  |  | From *.pid field on the first page of the CRF |
| PI_MAL | PI s Diagnosis of malaria | Char 2 | N = No |  | See Analysis plan |
|  |  |  | Y = Yes |  |  |
| PNEUM10_ | Primary case definition pneumonia | Num 8 |  |  | See Analysis plan |
| PNEUM21_ | Secondary case definition 1 pneumonia | Num 8 |  |  |  |
| PNEUM22_ | Secondary case definition 2 pneumonia | Num 8 |  |  |  |
| PNEUM23_ | Secondary case definition 2 pneumonia | Num 8 |  |  |  |
| POSS_MEN | Possible meningitis | Num 8 | 0 = No |  |  |
|  |  |  | 1 = Yes |  |  |
| PROSTRAT | Prostration | Num 8 |  |  | Derived: 1 if signs.prostrat=”Y”. 0 otherwise |
| REQ3RDS | Were three readings required? | Num 8 |  |  | See wslide.req3rds |
| RESPDIST | Respiratory distress | Num 8 |  |  | Derived: 1 if signs.lowchest=Y or signs.breath=Y. 0 otherwise |
| S1_DENS | Slide 1 density | Char 7 |  |  | See wslide.s1_dens  Raw parasite density read 1 associated with the selected a_pf_den from w_slide |
| S2_DENS | Slide 2 density | Char 7 |  |  | See wslide.s2_dens  Raw parasite density read 2 associated with the selected a_pf_den from w_slide |
| S3_DENS | Slide 3 density | Char 7 |  |  | See wslide.s3_dens  Raw parasite density read 3 associated with the selected a_pf_den from w_slide |
| SALM_SEP | Sepsis case def 2 Salmonella sepsis | Num 8 |  |  | See Analysis plan |
| SEIZURES | Seizures | Num 8 |  |  | Derived: |
| SEPSIS | Sepsis case definition 1 | Num 8 |  |  | See Analysis plan |
| SEV10_ | Severe malaria primary case definition | Num 8 |  |  | See Analysis plan |
| SEV21_ | Severe malaria secondary case def 1 | Num 8 |  |  |  |
| SEV22_ | Severe malaria secondary case def 2 | Num 8 |  |  |  |
| SEV23_ | Severe malaria secondary case def 3 | Num 8 |  |  |  |
| SEV_AN1_ | Incident severe anaemia case def 1 | Num 8 |  |  | See Analysis plan |
| SEV_AN2_ | Incident severe anaemia case def 2 | Num 8 |  |  |  |
| SEV_AN3_ | Incident severe anaemia case def 3 | Num 8 |  |  |  |

Study 110021 - wconvac

| **Variable** | **Description** | **Type** | **Codes** | **Crf Page** | **Comment** |
| --- | --- | --- | --- | --- | --- |
| ACTIVITY | Activity | Num 8 | 231 = CONC.VACC2 |  | From *.activity field on top of first page of each CRF segment |
|  |  |  | 51 = CONC.VACC1 |  |  |
| ACT_DESC | Activity Description | Char 7 | SECTION |  | From protocol. Label of the activity. |
| ASTER | Conc vacc in [-7, 30] days of vacc | Char 1 |  |  | Derived: Concomitant vaccine administered in the period beginning 7 days before up to 30 days after the date of administration of a study vaccine |
| CONVACID | Ocean Identificatin Nb | Num 8 |  |  | vacc_con.convacid ALL VALUES MISSING |
| L_ROUT | Route | Char 17 | INTRADERMAL |  | Derived: decoded value of (med_rout) |
|  |  |  | INTRAMUSCULAR |  |  |
|  |  |  | MISSING CONFIRMED |  |  |
|  |  |  | ORAL |  |  |
|  |  |  | OTHER |  |  |
|  |  |  | SUBCUTANEOUS |  |  |
| MEDSRDAT | Concomitant vaccination date | Char 9 |  | crf1:68, 110 | vacc_con.medsrdat  DDMMMYYYY format |
| MED_ROUT | Route | Char 3 | ID = INTRADERMAL | crf1:68, 110 | vacc_con.med_rout |
|  |  |  | IM = INTRAMUSCULAR |  |  |
|  |  |  | MC = MISSING CONFIRMED |  |  |
|  |  |  | OTH = OTHER |  |  |
|  |  |  | PO = ORAL |  |  |
|  |  |  | SC = SUBCUTANEOUS |  |  |
| MELASTDO | Last dose administered | Num 8 |  |  | Derived: last study dose administered before the administration of the concomitant vaccination |
| MENEXTDO | Next study dose admin after concadmin | Num 8 |  |  | Derived: next study dose administered after the administration of the concomitant vaccination. |
| ME_DAY_O | Day of onset | Num 8 |  |  | Derived: onset day of the administration relative to the last study vaccine dose administered (melastdo). 0=on the day of study dose administration. |
| ME_DNEXT | Num days before next study vaccination | Num 8 |  |  | Derived: number of days between the date of the concomitant vaccination and the date of the next study vaccine dose administered. |
| NUM_VIS | Date of visit | Num 8 |  |  | Derived: wdates.raw_date (where wdates.type_lst=’VIS’) in SAS date format, in case of administration of specific non-investigational study vaccines. Otherwise empty. ALL VALUES MISSING |
| PID | Subject Identification | Num 8 |  |  | From *.pid field on the first page of the CRF |
| STR_DATE | Concomitant vaccination date | Num 8 |  |  | Derived: (medsrdat) in SAS date format |
| TRADNAME | Trade name | Char 60 |  | crf1:68, 110 | vacc_con.tradname |
| VAC_D | Previous study vaccination date | Char 9 |  |  | Derived: latest date when a study vaccine dose was administered BEFORE or on the administration day of the concomitant vaccine (DD-MMM-YYYY). |
| VAC_NEXT | Next study vaccination date | Char 9 |  |  | Derived: earliest date when a study vaccine dose was administered AFTER the administration of the concomitant vaccine (DD-MMM-YYYY). |
| VIS_DATE | Date of visit | Char 9 |  |  | Derived: wdates.raw_date (where wdates.type_lst=’VIS’) in DD-MMM-YYYY format, in case of administration of specific non-investigational study vaccines. Otherwise empty. ALL VALUES MISSING |

Dataset limited to subjects with any vaccine administration reported, other than the study vaccine(s) (cvsheet.cv_flag=’Y’)

Study 110021 - wdates

| **Variable** | **Description** | **Type** | **Codes** | **Crf Page** | **Comment** |
| --- | --- | --- | --- | --- | --- |
| ACTIVITY | Activity | Num 8 | 10 = SCREENING (VISIT1) OR VISIT1 |  | From *.activity field on top of first page of each CRF segment |
|  |  |  | 161 = STATUS_V16 |  |  |
|  |  |  | 20 = PRE (VISIT2) OR VISIT2 |  |  |
|  |  |  | 220 = PIII(M20) (VISIT22) OR VISIT22 |  |  |
|  |  |  | 221 = STATUS_V22 |  |  |
|  |  |  | 230 = PIV(M21) (VISIT23) OR VISIT23 |  |  |
|  |  |  | 30 = VISIT3 |  |  |
|  |  |  | 340 = PIV(M32) (VISIT34) OR VISIT34 |  |  |
|  |  |  | 350 = VISIT35 |  |  |
|  |  |  | 360 = PIV(M44) (VISIT36) OR VISIT36 |  |  |
|  |  |  | 380 = PIV(M56) (VISIT38) OR VISIT38 |  |  |
|  |  |  | 40 = VISIT4 |  |  |
|  |  |  | 50 = PIII(M3) (VISIT5) OR VISIT5 |  |  |
|  |  |  | 940 = CONCLUS |  |  |
|  |  |  | 941 = CONCLUS3 |  |  |
| PID | Subject Identification | Num 8 |  |  | From *.pid field on the first page of the CRF |
| RAW_DATE | Date for typ(lst/int) | Char 9 |  | crf1:1, 25, 41, 43, 53, 55, 65, 91, 107, 125, 138, 140, 146 | actdates.actrdate  (Date of visits) |
|  |  |  |  | crf1:83, 104, 134, 152 | conclus.lc_rdat  (Last contact date) |
|  |  |  |  | crf1:1, 135 | consent.cons_dat  (Informed Consent Date) |
|  |  |  |  | crf1:1 | demog.dob_raw  (Date of birth) |
|  |  |  |  | crf1:3, 66, 93, 94, 108, 126, 127, 141, 142, 143, 147, 148, 149 | labsheet.samprdat  (Sample date) |
|  |  |  |  | crf1:30, 31, 42, 43, 54, 55, 95, 96 | v_info.vaccrdat  (Vaccination date when administration not during a visit) |
| RELVACDO | Activity related vaccine dose | Num 8 | . |  | From protocol. Vaccine dose planned at the activity in the protocol. So this dose number is directly related to the activity irrespective of whether the dose was administered or not. |
|  |  |  | 0 |  |  |
|  |  |  | 1 |  |  |
|  |  |  | 2 |  |  |
|  |  |  | 3 |  |  |
|  |  |  | 4 |  |  |
| SCHEDULE | Schedule (clinical effective) | Num 8 |  |  | See pid.schedule |
| SEQ_INT | Sequence for Interval | Num 8 |  |  | Derived: occurrences of events are numbered sequentially within subjects for each type of event, starting at 1, according to the schedule specified in the protocol. INTERNAL USE |
| SEQ_LST | Sequence for date_id | Num 8 |  |  | Derived: occurrences of events are numbered sequentially within subjects for each type of event, starting at 1, according to the schedule specified in the protocol. INTERNAL USE |
| TIT_INT | Title for interval | Char 30 | CONCLUS |  | Derived: Description of the event - short description |
|  |  |  | CONCLUS3 |  |  |
|  |  |  | DOB |  |  |
|  |  |  | Dose:1 |  |  |
|  |  |  | Dose:2 |  |  |
|  |  |  | Dose:3 |  |  |
|  |  |  | Dose:4 |  |  |
|  |  |  | Inf Cons1 |  |  |
|  |  |  | Inf Cons2 |  |  |
|  |  |  | Last date*of contact |  |  |
|  |  |  | PIII(M20) |  |  |
|  |  |  | PIII(M3) |  |  |
|  |  |  | PIV(M21) |  |  |
|  |  |  | PIV(M32) |  |  |
|  |  |  | PIV(M44) |  |  |
|  |  |  | PIV(M56) |  |  |
|  |  |  | PRE |  |  |
|  |  |  | SCREENING |  |  |
|  |  |  | STATUS_V16 |  |  |
|  |  |  | STATUS_V22 |  |  |
|  |  |  | VIS?? 10 |  |  |
|  |  |  | VISIT1 |  |  |
|  |  |  | VISIT2 |  |  |
|  |  |  | VISIT22 |  |  |
|  |  |  | VISIT23 |  |  |
|  |  |  | VISIT3 |  |  |
|  |  |  | VISIT34 |  |  |
|  |  |  | VISIT35 |  |  |
|  |  |  | VISIT36 |  |  |
|  |  |  | VISIT38 |  |  |
|  |  |  | VISIT4 |  |  |
|  |  |  | VISIT5 |  |  |
|  |  |  | activity 10 |  |  |
| TIT_LST | Title for Date_id | Char 50 | CONCLUS |  | Derived: Description of the event - long description |
|  |  |  | CONCLUS3 |  |  |
|  |  |  | Dates* of *birth |  |  |
|  |  |  | Informed date |  |  |
|  |  |  | Last date*of contact |  |  |
|  |  |  | STATUS_V16 |  |  |
|  |  |  | STATUS_V22 |  |  |
|  |  |  | VIS ---*activity 10 |  |  |
|  |  |  | VIS?? 10 |  |  |
|  |  |  | VISIT1 |  |  |
|  |  |  | VISIT1*SCREENING |  |  |
|  |  |  | VISIT2 |  |  |
|  |  |  | VISIT2*Dose:1 |  |  |
|  |  |  | VISIT2*PRE |  |  |
|  |  |  | VISIT22 |  |  |
|  |  |  | VISIT22*Dose:4 |  |  |
|  |  |  | VISIT22*PIII(M20) |  |  |
|  |  |  | VISIT23 |  |  |
|  |  |  | VISIT23*PIV(M21) |  |  |
|  |  |  | VISIT3 |  |  |
|  |  |  | VISIT3*Dose:2 |  |  |
|  |  |  | VISIT34 |  |  |
|  |  |  | VISIT34*PIV(M32) |  |  |
|  |  |  | VISIT35 |  |  |
|  |  |  | VISIT36 |  |  |
|  |  |  | VISIT36*PIV(M44) |  |  |
|  |  |  | VISIT38 |  |  |
|  |  |  | VISIT38*PIV(M56) |  |  |
|  |  |  | VISIT4 |  |  |
|  |  |  | VISIT4*Dose:3 |  |  |
|  |  |  | VISIT5 |  |  |
|  |  |  | VISIT5*PIII(M3) |  |  |
| TYPE_INT | Type for interval | Char 6 | BL = Date of blood sample |  | From data management meta-data. Each type of events receives a code during set-up of the internal databases. |
|  |  |  | BL1 |  |  |
|  |  |  | BL2 |  |  |
|  |  |  | BL3 |  |  |
|  |  |  | CON = Date of last contact |  |  |
|  |  |  | DOB = Date of birth |  |  |
|  |  |  | HE = Date of sampling for hematology |  |  |
|  |  |  | INF = Informed consent date |  |  |
|  |  |  | VAC = Vaccination date |  |  |
|  |  |  | VIS = Date of visits |  |  |
| TYPE_LST | Type for date_id | Char 6 | BL = Date of blood sample |  | From data management meta-data. Each type of events receives a code during set-up of the internal databases. |
|  |  |  | BL1 |  |  |
|  |  |  | BL2 |  |  |
|  |  |  | BL3 |  |  |
|  |  |  | CON = Date of last contact |  |  |
|  |  |  | DOB = Date of birth |  |  |
|  |  |  | HE = Date of sampling for hematology |  |  |
|  |  |  | INF = Informed consent date |  |  |
|  |  |  | VAC = Vaccination date |  |  |
|  |  |  | VIS = Date of visits |  |  |

Study 110021 - wdemog

| **Variable** | **Description** | **Type** | **Codes** | **Crf Page** | **Comment** |
| --- | --- | --- | --- | --- | --- |
| ACTIVITY | Activity | Num 8 | 10 = SCREENING (VISIT1) OR VISIT1 |  | From *.activity field on top of first page of each CRF segment |
| ACT_DE2 | Activity Description (2) | Char 15 | VISIT2 |  | From protocol. Label of the activity. |
| ACT_DE3 | Activity Description (3) | Char 15 | VISIT22 |  | From protocol. Label of the activity. |
| ACT_DE4 | Activity Description (4) | Char 15 | VISIT22 |  | From protocol. Label of the activity. |
| ACT_DESC | Activity Description | Char 15 | VISIT2 |  | From protocol. Label of the activity. |
| ACT_R2 | Ref activity number for age (2) | Num 8 | 20 = PRE (VISIT2) OR VISIT2 |  | From analysis plan. Reference activity for computing the age (2) |
| ACT_R3 | Ref activity number for age (3) | Num 8 | 220 = PIII(M20) (VISIT22) OR VISIT22 |  | From analysis plan. Reference activity for computing the age (3) |
| ACT_R4 | Ref activity number for age (4) | Num 8 | 220 = PIII(M20) (VISIT22) OR VISIT22 |  | From analysis plan. Reference activity for computing the age (4) |
| ACT_REF | Ref activity number for age | Num 8 | 20 = PRE (VISIT2) OR VISIT2 |  | From analysis plan. Reference activity for computing the age |
| AGE | Age (M) | Num 8 |  |  | Derived: Age at the reference activity, computed as the number of units (see wdemog.age_unit) between wdemog.dob_raw and wdemog.date_ref |
| AGE2 | Age (W) (2) | Num 8 |  |  | Derived: Age at the reference activity, computed as the number of units (see wdemog.age_un2) between wdemog.dob_raw and wdemog.date_r2 |
| AGE3 | Age (M) (3) | Num 8 |  |  | Derived: Age at the reference activity,computed as the number of units (see w_demog.age_un3) between w_demog.dob_raw and w_demog.date_r3 |
| AGE4 | Age (W) (4) | Num 8 |  |  | Derived: Age at the reference activity,computed as the number of units (see w_demog.age_un4) between w_demog.dob_raw and w_demog.date_r4 |
| AGEC | Age Group (N) | Num 8 | 1 = [5-17] months |  | See pid.agec |
|  |  |  | 2 = [6-12] weeks |  |  |
| AGE_CAT | AGE_CAT | Char 14 |  |  |  |
|  |  |  | [5-11] months |  |  |
|  |  |  | [12-17] months |  |  |
| AGE_UN2 | Age unit (2) | Char 1 | W = week |  | From analysis plan |
| AGE_UN3 | Age unity (3) | Char 1 | M = month |  | From analysis plan |
| AGE_UN4 | Age unity (4) | Char 1 | W = week |  | From analysis plan |
| AGE_UNIT | Age unit | Char 1 | M = month |  | From analysis plan |
| BEDNT_ | BEDNT_ | Char 6 |  |  |  |
|  |  |  | ITN |  |  |
|  |  |  | No ITN |  |  |
| BEDNT_M14 | BEDNT_M14 | Char 6 |  |  |  |
| BEDNT_M31 | BEDNT_M31 | Char 6 |  |  |  |
| BL_SP_2 | Timepoint of BS (2) | Char 1 |  |  | See evtlab.bl_sp_sc   ALL VALUES MISSING |
| BL_SP_3 | Timepoint of BS | Char 1 |  |  | ALL VALUES MISSING |
| BL_SP_4 | Timepoint of BS (2) | Char 1 |  |  | ALL VALUES MISSING |
| BL_SP_SC | Timepoint of BS | Char 1 |  |  | See evtlab.bl_sp_sc   ALL VALUES MISSING |
| BSLAN | BSLAN | Char 1 | N = No |  |  |
|  |  |  | Y = Yes |  |  |
| BSLDINP | BSLDINP | Num 8 |  |  |  |
| BSLDOUT | BSLDOUT | Num 8 |  |  |  |
| BSLDOUTC | BSLDOUTC | Char 6 | <=5 km |  |  |
|  |  |  | >5 km |  |  |
| BSLHB | BSLHB | Num 8 |  |  |  |
| CENTER | Center number | Char 10 |  |  | demog.center |
| COD_RACE | Codelist for race | Char 20 | RACE_V122 |  | Derived: For internal use |
| CSTRTL_PD3 | CSTRTL_PD3 | Num 8 |  |  |  |
| CSTRTL_PD4 | CSTRTL_PD4 | Num 8 |  |  |  |
| DATE_R2 | Reference date for computing age (2) | Char 9 |  | crf1:1, 25, 41, 43, 53, 55, 65, 91, 107, 125, 138, 140, 146 | actdates.actrdate  (for visit dates) |
|  |  |  |  | crf1:1, 135 | consent.cons_dat  (for inform consent date) |
|  |  |  |  | crf1:3, 66, 93, 94, 108, 126, 127, 141, 142, 143, 147, 148, 149 | labsheet.samprdat  (for sampling dates) |
|  |  |  |  | crf1:30, 31, 42, 43, 54, 55, 95, 96 | v_info.vaccrdat  (for vaccination dates when administration not during a visit) |
| DATE_R3 | Reference date for computing age (3) | Char 9 |  | crf1:1, 25, 41, 43, 53, 55, 65, 91, 107, 125, 138, 140, 146 | actdates.actrdate  (for visit dates) |
|  |  |  |  | crf1:1, 135 | consent.cons_dat  (for inform consent date) |
|  |  |  |  | crf1:3, 66, 93, 94, 108, 126, 127, 141, 142, 143, 147, 148, 149 | labsheet.samprdat  (for sampling dates) |
|  |  |  |  | crf1:30, 31, 42, 43, 54, 55, 95, 96 | v_info.vaccrdat  (for vaccination dates when administration not during a visit) |
| DATE_R4 | Reference date for computing age (4) | Char 9 |  | crf1:1, 25, 41, 43, 53, 55, 65, 91, 107, 125, 138, 140, 146 | actdates.actrdate  (for visit dates) |
|  |  |  |  | crf1:1, 135 | consent.cons_dat  (for inform consent date) |
|  |  |  |  | crf1:3, 66, 93, 94, 108, 126, 127, 141, 142, 143, 147, 148, 149 | labsheet.samprdat  (for sampling dates) |
|  |  |  |  | crf1:30, 31, 42, 43, 54, 55, 95, 96 | v_info.vaccrdat  (for vaccination dates when administration not during a visit) |
| DATE_REF | Reference date for computing age | Char 9 |  | crf1:1, 25, 41, 43, 53, 55, 65, 91, 107, 125, 138, 140, 146 | actdates.actrdate  (for visit dates) |
|  |  |  |  | crf1:1, 135 | consent.cons_dat  (for inform consent date) |
|  |  |  |  | crf1:3, 66, 93, 94, 108, 126, 127, 141, 142, 143, 147, 148, 149 | labsheet.samprdat  (for sampling dates) |
|  |  |  |  | crf1:30, 31, 42, 43, 54, 55, 95, 96 | v_info.vaccrdat  (for vaccination dates when administration not during a visit) |
| DOB_RAW | Date of Birth | Char 9 |  | crf1:1 | demog.dob_raw |
| ENDITT_EE | End date of FU period for cohort ITT | Num 8 |  |  | End of ITT [M0-Dec13] |
| ENDITT_SE | End date of FU period for cohort ITT | Num 8 |  |  | End of ITT [M0-M32] |
| ETHNIC | Ethnicity | Char 2 |  |  | NOT APPLICABLE |
| EXTENSION | EXTENSION | Num 8 |  |  | Consented to extension phase (M32-Dec13) |
| GEST_FLG | Baby born >= 37th week of gestation | Char 3 | N = No |  |  |
|  |  |  | Y = Yes |  |  |
| GEST_WK | Duration of gestation in weeks at birth | Char 2 |  |  |  |
| HAZ | Length/height-for-age z-score | Num 8 |  |  |  |
| INP1 | Distance/Inpatient/Facility/(KM) at V2 | Char 5 |  |  |  |
| INP2 | Distance/Inpatient/Facility/(KM) at V22 | Char 5 |  |  |  |
| M20AN | Anemia at M20 | Char 1 | N = No |  |  |
|  |  |  | Y = Yes |  |  |
| M20HAZ | Length/height-for-age z-score | Num 8 |  |  |  |
| M20HB | Hb value at M20 | Num 8 |  |  |  |
| M20MUACZ | Arm circumference-for-age z score | Num 8 |  |  |  |
| M20WAZ | Weight-for-age z-score | Num 8 |  |  |  |
| M20WHZ | Weight-for-length/height z-score | Num 8 |  |  |  |
| MUACZ | Arm circumference-for-age z score | Num 8 |  |  |  |
| OUT1 | Distance/Outpatient/Facility/(KM) at V2 | Char 5 |  |  |  |
| OUT2 | Distance/Outpatient/Facility/(KM) at V22 | Char 5 |  |  |  |
| PID | Subject Identification | Num 8 |  |  | From *.pid field on the first page of the CRF |
| PREV_SAE | SAE before visit 1? | Char 2 |  |  | ALL VALUES MISSING |
| PR_PID | Previous Subject No | Num 8 |  |  | NOT APPLICABLE |
| PR_ST_ID | Previous study number | Char 15 |  |  | Derived: Creation from old nomenclature : pr_st_nb/pr_cpms ALL VALUES MISSING |
| RACE | Race | Char 2 | 1 = AFRICAN HERITAGE / AFRICAN AMERICAN | crf1:1 | demog.race |
| RACE_DEC | Race decoded | Char 41 | African Heritage / African American |  | Derived: decoded value of (race) |
| RACE_NEW | Label Race | Char 60 | AFRICAN HERITAGE / AFRICAN AMERICAN |  | Derived: (race_oth) if specified, else decoded value of (race). |
| RACE_OTH | Race - specification | Char 40 |  | crf1:1 | demog.race_oth ALL VALUES MISSING |
| RELVAC2 | Activity related vaccine dose | Num 8 |  |  | From protocol. Vaccine dose planned at the activity in the protocol. So this dose number is directly related to the activity irrespective of whether the dose was administered or not. |
| RELVAC3 | Relative vaccine dose | Num 8 |  |  | ALL VALUES MISSING |
| RELVAC4 | Relative vaccine dose (2) | Num 8 |  |  | ALL VALUES MISSING |
| RELVACDO | Activity related vaccine dose | Num 8 | 1 |  | From protocol. Vaccine dose planned at the activity in the protocol. So this dose number is directly related to the activity irrespective of whether the dose was administered or not. |
| SCHEDULE | Schedule (clinical effective) | Num 8 |  |  | See pid.schedule |
| SCH_SCR | Schedule (screening phase) | Num 8 |  |  | NOT APPLICABLE |
| SEX | Gender | Char 2 | F = FEMALE | crf1:1 | demog.sex |
|  |  |  | M = MALE |  |  |
| SITE | Site | Char 9 | Agogo |  | See pid.site |
|  |  |  | Bagamoyo |  |  |
|  |  |  | Kilifi |  |  |
|  |  |  | Kintampo |  |  |
|  |  |  | Kombewa |  |  |
|  |  |  | Korogwe |  |  |
|  |  |  | Lambarene |  |  |
|  |  |  | Lilongwe |  |  |
|  |  |  | Manhica |  |  |
|  |  |  | Nanoro |  |  |
|  |  |  | Siaya |  |  |
| STRTITT | Start date of FU period for cohort ITT | Num 8 |  |  | Start of ITT [M0-Dec13] |
| TRIBE | Tribe | Char 20 | Adas = ADAS | crf1:1 | demog.tribe |
|  |  |  | Akans = AKANS |  |  |
|  |  |  | Akele = AKELE |  |  |
|  |  |  | Bimoba = BIMOBA |  |  |
|  |  |  | Bonos = BONOS |  |  |
|  |  |  | Builsas = BUILSAS |  |  |
|  |  |  | Busangas = BUSANGAS |  |  |
|  |  |  | Changanas = CHANGANAS |  |  |
|  |  |  | Chewa = CHEWA |  |  |
|  |  |  | Chonyi = CHONYI |  |  |
|  |  |  | Dagartis = DAGARTIS |  |  |
|  |  |  | Dagombas = DAGOMBAS |  |  |
|  |  |  | Doe = DOE |  |  |
|  |  |  | Ewes = EWES |  |  |
|  |  |  | Fang = FANG |  |  |
|  |  |  | Frafra = FRAFRA |  |  |
|  |  |  | Fulani = FULANI |  |  |
|  |  |  | Gas = GAS |  |  |
|  |  |  | Giriama = GIRIAMA |  |  |
|  |  |  | Gishira-Punu = GISHIRA-PUNU |  |  |
|  |  |  | Gonjas = GONJAS |  |  |
|  |  |  | Gourounssi = GOUROUNSSI |  |  |
|  |  |  | Grumah = GRUMAH |  |  |
|  |  |  | Grushie = GRUSHIE |  |  |
|  |  |  | Ikota = IKOTA |  |  |
|  |  |  | Jibana = JIBANA |  |  |
|  |  |  | Kanjaga = KANJAGA |  |  |
|  |  |  | Kisii = KISII |  |  |
|  |  |  | Krobos = KROBOS |  |  |
|  |  |  | Kusasi = KUSASI |  |  |
|  |  |  | Kwere = KWERE |  |  |
|  |  |  | Lambiya = LAMBIYA |  |  |
|  |  |  | Lomwe = LOMWE |  |  |
|  |  |  | Luhya = LUHYA |  |  |
|  |  |  | Luo = LUO |  |  |
|  |  |  | Makonde = MAKONDE |  |  |
|  |  |  | Mamprusi = MAMPRUSI |  |  |
|  |  |  | Mang anja = MANG ANJA |  |  |
|  |  |  | Mbondei = MBONDEI |  |  |
|  |  |  | Mchaga = MCHAGA |  |  |
|  |  |  | Mfipa = MFIPA |  |  |
|  |  |  | Mngoni = MNGONI |  |  |
|  |  |  | Mossi = MOSSI |  |  |
|  |  |  | Mpare = MPARE |  |  |
|  |  |  | Msambaa = MSAMBAA |  |  |
|  |  |  | Muha = MUHA |  |  |
|  |  |  | Myene = MYENE |  |  |
|  |  |  | Mzigua = MZIGUA |  |  |
|  |  |  | Nankana = NANKANA |  |  |
|  |  |  | Ngoni = NGONI |  |  |
|  |  |  | Nkhonde = NKHONDE |  |  |
|  |  |  | Nsenga = NSENGA |  |  |
|  |  |  | Nzebi = NZEBI |  |  |
|  |  |  | Other = OTHER |  |  |
|  |  |  | Rongas = RONGAS |  |  |
|  |  |  | Sena = SENA |  |  |
|  |  |  | Sissalas = SISSALAS |  |  |
|  |  |  | Tsogo = TSOGO |  |  |
|  |  |  | Tumbuka = TUMBUKA |  |  |
|  |  |  | Walas = WALAS |  |  |
|  |  |  | Yawo = YAWO |  |  |
|  |  |  | Zaramo = ZARAMO |  |  |
|  |  |  | Zigua = ZIGUA |  |  |
| TYPE_R2 | Activity Type | Char 3 | VAC = Vaccination Date |  | From protocol. Type of event used for deriving age (2). See wdemog.date_r2 |
| TYPE_R3 | Type date used as reference for age (3) | Char 3 | VIS = Visit Date |  | From protocol. Type of event used for deriving age (3). See wdemog.date_r3 |
| TYPE_R4 | Type date used as reference for age (4) | Char 3 | VIS = Visit Date |  | From protocol. Type of event used for deriving age (4). See wdemog.date_r4 |
| TYPE_REF | Activity Type | Char 3 | VAC = Vaccination Date |  | From protocol. Type of event used for deriving age. See wdemog.date_ref |
| WAZ | Weight-for-age z-score | Num 8 |  |  |  |
| WHZ | Weight-for-length/height z-score | Num 8 |  |  |  |

Study 110021 - wgrow

| **Variable** | **Description** | **Type** | **Codes** | **Crf Page** | **Comment** |
| --- | --- | --- | --- | --- | --- |
| ACTIVITY | Activity | Num 8 | 20 = VISIT2 |  | From *.activity field on top of first page of each CRF segment |
|  |  |  | 220 = VISIT22 |  |  |
|  |  |  | 340 = VISIT34 |  |  |
|  |  |  | 360 = VISIT36 |  |  |
|  |  |  | 380 = VISIT38 |  |  |
|  |  |  | 50 = VISIT5 |  |  |
| ACT_DESC | Activity Description | Char 15 | VISIT2 |  | From protocol. Label of the activity. |
|  |  |  | VISIT22 |  |  |
|  |  |  | VISIT34 |  |  |
|  |  |  | VISIT36 |  |  |
|  |  |  | VISIT38 |  |  |
|  |  |  | VISIT5 |  |  |
| AGE | Age (D) | Num 8 |  |  | See wdemog.age |
| AGEDAYS | Age in days | Num 8 |  |  | Derived: calulated age in days for deriving z-score |
| BMIZ | BMI-for-age z-score | Num 8 |  |  | Derived: |
| BMIZF | Flag for bmiz<-5 or bmiz>5 | Num 8 |  |  | Derived:1 if bmiz<-5 or bmiz>5, 0 if -5<=bmiz<=5, Null otherwise |
| CBMI | Calulated BMI=weight / squared(CLENHEI) | Num 8 |  |  | Derived: |
| CLENHEI | Converted length/height (cm) | Num 8 |  |  | Derived: Converted length/height (cm) for deriving z-score |
| HAZ | Length/height-for-age z-score | Num 8 |  |  | Derived: |
| HAZF | Flag for haz<-6 or haz>6 | Num 8 |  |  | Derived: 1 if haz<-6 or haz>6, 0 if -6<=haz<=6, null otherwise |
| HEI_CM | Height in cm | Num 8 |  |  |  |
| MUACZ | Arm circumference-for-age z score | Num 8 |  |  | Derived: |
| MUACZF | Flag for muacz<-5 or muacz>5 | Num 8 |  |  | Derived: 1 if muacz<-5 or muacz>5, 0 if -5<=muacz<=5, null otherwise |
| MUAC_CM | MUAC_CM | Num 8 |  |  |  |
| PID | Subject Identification | Num 8 |  |  | From *.pid field on the first page of the CRF |
| SEX | Gender | Char 2 | F = Female |  | See wdemog.sex |
|  |  |  | M = Male |  |  |
| WAZ | Weight-for-age z-score | Num 8 |  |  | Derived: |
| WAZF | Flag for waz<-6 or waz>5 | Num 8 |  |  | Derived: 1 if waz<-6 or waz>5, 0 if -6<=waz<=5, null otherwise |
| WEI_KG | Weight in kg | Num 8 |  |  |  |
| WHZ | Weight-for-length/height z-score | Num 8 |  |  | Derived: |
| WHZF | Flag for whz<-5 or whz>5 | Num 8 |  |  | Derived: 1 if whz<-5 or whz>5, 0 if -5<=whz<=5 |

Study 110021 - wicd10

| **Variable** | **Description** | **Type** | **Codes** | **Crf Page** | **Comment** |
| --- | --- | --- | --- | --- | --- |
| ACTIVITY | Activity | Num 8 | 40 = VISIT4 |  | From *.activity field on top of first page of each CRF segment |
|  |  |  | 50 = VISIT5 |  |  |
| DEATHDC1 | DEATHDC1 | Char 250 |  |  |  |
| DEATHDC2 | DEATHDC2 | Char 250 |  |  |  |
| DEATHDC3 | DEATHDC3 | Char 250 |  |  |  |
| DEATHDC4 | DEATHDC4 | Char 250 |  |  |  |
| DEATHDC5 | DEATHDC5 | Char 250 |  |  |  |
| DEATHDC6 | DEATHDC6 | Char 250 |  |  | ALL VALUES MISSING |
| DEATHDC7 | DEATHDC7 | Char 250 |  |  | ALL VALUES MISSING |
| DEATHDC8 | DEATHDC8 | Char 250 |  |  | ALL VALUES MISSING |
| DEATH_C1 | Death cause code 1 - ICD-10 | Char 7 |  |  |  |
| DEATH_C2 | Death cause code 2 - ICD-10 | Char 7 |  |  |  |
| DEATH_C3 | Death cause code 3 - ICD-10 | Char 7 |  |  |  |
| DEATH_C4 | Death cause code 4 - ICD-10 | Char 7 |  |  |  |
| DEATH_C5 | Death cause code 5 - ICD-10 | Char 7 |  |  |  |
| DEATH_C6 | Death cause code 6 - ICD-10 | Char 7 |  |  | ALL VALUES MISSING |
| DEATH_C7 | Death cause code 7 - ICD-10 | Char 7 |  |  | ALL VALUES MISSING |
| DEATH_C8 | Death cause code 8 - ICD-10 | Char 7 |  |  | ALL VALUES MISSING |
| DEATH_DT | Date of Death | Char 9 |  |  |  |
| DOD_NUM | Date of death (numeric) | Num 8 |  |  |  |
| ENDITT | End date of FU period for cohort ITT | Num 8 |  |  |  |
| LIST_CD | List of ICD-10 codes | Char 56 |  |  |  |
| LIST_DC | List of decoded ICD-10 codes | Char 2000 |  |  |  |
| PID | Subject Identification | Num 8 |  |  | From *.pid field on the first page of the CRF |
| STRTITT | Start date of FU period for cohort ITT | Num 8 |  |  |  |

Study 110021 - wmedic

| **Variable** | **Description** | **Type** | **Codes** | **Crf Page** | **Comment** |
| --- | --- | --- | --- | --- | --- |
| ACTIVITY | Activity | Num 8 | 231 = MEDIC2 |  | From *.activity field on top of first page of each CRF segment |
|  |  |  | 385 = MEDIC3 |  |  |
|  |  |  | 500 = MED SERIOUS |  |  |
|  |  |  | 51 = MEDIC1 |  |  |
| ANTIPYR | Antipyretic flag | Char 1 | Y = antipyretic as per GSK drug dictionary |  | Derived: flag set to ‘Y’ by automatic encoder if the medication is classified as an antipyretic in the GSK drug dictionary. |
| ASTER | Medic 7 bef or 30 after vacc [inc] | Char 1 | * = medication was taken within day -7 & day 30 of vaccination |  | Derived: Concomitant medication taken in the period beginning 7 days before up to 30 days after the date of administration of a study vaccine. |
| END_DATE | End of medication | Num 8 |  |  | Derived: (mederdat) in SAS date format |
| GSK_COD | GSK drug code of the medication | Char 8 |  |  | Derived: code of the product in the GSK drug dictionary matching the reported product name (tradname). Matching is performed by an automatic encoder. When the automatic encoder could not find a match, a physician standardized the description (tradname) into (gsk_mod). Then the automatic encoder assigned the code using (gsk_mod) for the match. |
| GSK_FLAG | Auto encoding flag | Char 2 |  |  | Derived: flag assigned by the auto-encoder. FOR INTERNAL USE ONLY. |
| GSK_MOD | Trade name modified (for GSK drug) | Char 60 |  |  | From physician. When the verbatim of the product (tradname) could not be matched to an entry of the GSK drug dictionary by the automatic encoder, a physician assigned a standardized description - matching entries in the dictionary - manually. See (gsk_cod). |
| L_ROUT | Route | Char 17 | BOTH EARS |  | Derived: (med_rout) (decoded) |
|  |  |  | BOTH EYES |  |  |
|  |  |  | BUCAL |  |  |
|  |  |  | EXTERNAL |  |  |
|  |  |  | INHALATION |  |  |
|  |  |  | INTRADERMAL |  |  |
|  |  |  | INTRAMUSCULAR |  |  |
|  |  |  | INTRANASAL |  |  |
|  |  |  | INTRAOCULAR |  |  |
|  |  |  | INTRATHECAL |  |  |
|  |  |  | INTRAVENOUS |  |  |
|  |  |  | LEFT EAR |  |  |
|  |  |  | LEFT EYE |  |  |
|  |  |  | MISSING CONFIRMED |  |  |
|  |  |  | OPHTHALMIC |  |  |
|  |  |  | ORAL |  |  |
|  |  |  | OTHER |  |  |
|  |  |  | PARENTERAL |  |  |
|  |  |  | PER RECTUM |  |  |
|  |  |  | RECTAL |  |  |
|  |  |  | RIGHT EAR |  |  |
|  |  |  | RIGHT EYE |  |  |
|  |  |  | SUBCUTANEOUS |  |  |
|  |  |  | TOPICAL |  |  |
|  |  |  | UNKNOWN |  |  |
|  |  |  | VAGINAL |  |  |
| MD_TRANS | Translation | Char 60 |  | crf1:11, 69, 111, 151, 169 | medic.md_trans |
| MEDEND | End date DD-MM-YYYY | Char 11 |  |  | Derived: (mederdat)  in DD-MMM-YYYY format |
| MEDERDAT | Stop Date | Char 9 |  | crf1:11, 69, 111, 151, 169 | medic.mederdat |
| MEDINDIC | Indication | Char 80 |  | crf1:11, 69, 111, 151, 169 | medic.medindic |
| MEDSRDAT | Medication Start Date | Char 9 |  | crf1:11, 69, 111, 151, 169 | medic.medsrdat |
| MEDSTART | Medication Start Date(DD-MM-YYYY) | Char 11 |  |  | Derived: (medsrdat)  in DD-MMM-YYYY format |
| MED_DUR | Duration | Num 8 |  |  | Derived: Medication duration expressed in days and calculated as follows : (mederdat) - (medsrdat) + 1day |
| MED_ROUT | Medication Start route | Char 3 | AD = RIGHT EAR | crf1:11, 69, 111, 151, 169 | medic.med_rout |
|  |  |  | AS = LEFT EAR |  |  |
|  |  |  | AU = BOTH EARS |  |  |
|  |  |  | BU = BUCAL |  |  |
|  |  |  | EXT = EXTERNAL |  |  |
|  |  |  | ID = INTRADERMAL |  |  |
|  |  |  | IH = INHALATION |  |  |
|  |  |  | IM = INTRAMUSCULAR |  |  |
|  |  |  | IN = INTRANASAL |  |  |
|  |  |  | IO = INTRAOCULAR |  |  |
|  |  |  | IT = INTRATHECAL |  |  |
|  |  |  | IV = INTRAVENOUS |  |  |
|  |  |  | MC = MISSING CONFIRMED |  |  |
|  |  |  | NA = INTRANASAL |  |  |
|  |  |  | OO = RIGHT EYE |  |  |
|  |  |  | OP = OPHTHALMIC |  |  |
|  |  |  | OS = LEFT EYE |  |  |
|  |  |  | OTH = OTHER |  |  |
|  |  |  | OU = BOTH EYES |  |  |
|  |  |  | PA = PER RECTUM |  |  |
|  |  |  | PE = PARENTERAL |  |  |
|  |  |  | PO = ORAL |  |  |
|  |  |  | PR = RECTAL |  |  |
|  |  |  | SC = SUBCUTANEOUS |  |  |
|  |  |  | TO = TOPICAL |  |  |
|  |  |  | UNK = UNKNOWN |  |  |
|  |  |  | VA = VAGINAL |  |  |
| MELASTDO | Last dose administered | Num 8 |  |  | Derived: last study dose administered before or on the start of the concomitant medication |
| ME_DAY_O | Day of onset | Num 8 |  |  | Derived: onset day of the start of the medication relative to the last study dose administered (melastdo). 0=on the day of study dose administration. |
| PID | Subject Identification | Num 8 |  |  | From *.pid field on the first page of the CRF |
| PROPH_CK | Prophylactic | Char 2 | Y = Prophylactic medical indication | crf1:69, 111 | medic.proph_ck |
| STR_DATE | Medication Start Date(num value) | Num 8 |  |  | Derived: (medsrdat)  in SAS date format |
| TOT_DDOS | Total Daily Dose | Char 20 |  | crf1:69, 111, 151 | medic.tot_ddos |
| TRADNAME | Drug | Char 60 |  | crf1:11, 69, 111, 151, 169 | medic.tradname |
| VAC_DATE | Vaccination date | Num 8 |  |  | Derived: latest date when a study vaccine dose was administered BEFORE or on the start date of the medication (in SAS date format). |

Dataset limited to subjects with any medications/treatments administration reported (mdsheet.md_flag=’Y’)

Study 110021 - wmortal

| **Variable** | **Description** | **Type** | **Codes** | **Crf Page** | **Comment** |
| --- | --- | --- | --- | --- | --- |
| AGEC | Age Group (N) | Num 8 | 1 = [5-17] months |  | Derived: if 5<=age<= 17 months then AGEC=1; if 6<=age<= 12 weeks then AGEC=2 |
|  |  |  | 2 = [6-12] weeks |  |  |
| AGE_LABEL | Age Group | Char 13 |  |  | Derived: if 5<=age<= 17 months then AGE_LABEL=”[5-17] months”; if 6<=age<= 12 weeks then AGE_LABEL=”[6-12] weeks” |
| CENTER | Center number | Char 10 |  |  | See pid.center |
| CTY_COD | Country code | Char 3 | BF = Burkina Faso |  | ctr_info.cty_cod |
|  |  |  | GA = Gabon |  |  |
|  |  |  | GH = Ghana |  |  |
|  |  |  | KE = Kenya |  |  |
|  |  |  | MW = Malawi |  |  |
|  |  |  | MZ = Mozambique |  |  |
|  |  |  | TZ = Tanzania |  |  |
| CTY_NAM | Country name | Char 30 | Burkina Faso |  | ctr_info.cty_nam |
|  |  |  | Gabon |  |  |
|  |  |  | Ghana |  |  |
|  |  |  | Kenya |  |  |
|  |  |  | Malawi |  |  |
|  |  |  | Mozambique |  |  |
|  |  |  | Tanzania |  |  |
| D1 | Dose 1 date | Char 9 |  |  |  |
| DOD | Date of death | Char 9 |  |  |  |
| ELIG_CE | Eligibility(CE) | Char 1 | E = subject eliminated from the ATP cohort for safety and the ATP cohort for immunogenicity for elimination type |  | See Elim codes overview |
|  |  |  | I = subject eliminated from the ATP cohort for immunogenicity but not from the ATP cohort for safety for elimination type |  |  |
| ELIG_CM | Eligibility(CM) | Char 1 | E = subject eliminated from the ATP cohort for safety and the ATP cohort for immunogenicity for elimination type |  |  |
|  |  |  | I = subject eliminated from the ATP cohort for immunogenicity but not from the ATP cohort for safety for elimination type |  |  |
| ELIG_HS | Eligibility(HS) | Char 1 | I = subject eliminated from the ATP cohort for immunogenicity but not from the ATP cohort for safety for elimination type |  |  |
| ELIG_HV | Eligibility(HV) | Char 1 | E = subject eliminated from the ATP cohort for safety and the ATP cohort for immunogenicity for elimination type |  |  |
|  |  |  | I = subject eliminated from the ATP cohort for immunogenicity but not from the ATP cohort for safety for elimination type |  |  |
| ELIG_IE | Eligibility(IE) | Char 1 | E = subject eliminated from the ATP cohort for safety and the ATP cohort for immunogenicity for elimination type |  |  |
|  |  |  | I = subject eliminated from the ATP cohort for immunogenicity but not from the ATP cohort for safety for elimination type |  |  |
| ELIG_IM | Eligibility(IM) | Char 1 | E = subject eliminated from the ATP cohort for safety and the ATP cohort for immunogenicity for elimination type |  |  |
|  |  |  | I = subject eliminated from the ATP cohort for immunogenicity but not from the ATP cohort for safety for elimination type |  |  |
| ELIG_PT | Eligibility(PT) | Char 1 | E = subject eliminated from the ATP cohort for safety and the ATP cohort for immunogenicity for elimination type |  |  |
|  |  |  | I = subject eliminated from the ATP cohort for immunogenicity but not from the ATP cohort for safety for elimination type |  |  |
| ELIG_RI | Eligibility(RI) | Char 1 | E = subject eliminated from the ATP cohort for safety and the ATP cohort for immunogenicity for elimination type |  |  |
|  |  |  | I = subject eliminated from the ATP cohort for immunogenicity but not from the ATP cohort for safety for elimination type |  |  |
| ELIG_SM | Eligibility(SM) | Char 1 | E = subject eliminated from the ATP cohort for safety and the ATP cohort for immunogenicity for elimination type |  |  |
|  |  |  | I = subject eliminated from the ATP cohort for immunogenicity but not from the ATP cohort for safety for elimination type |  |  |
| ELIM_RCE | Elim reacto(CE) | Num 8 | 1 = subject eliminated from the ATP cohort for safety for elimination type |  |  |
| ELIM_RCM | Elim reacto(CM) | Num 8 | 1 = subject eliminated from the ATP cohort for safety for elimination type |  |  |
| ELIM_RHS | Elim reacto(HS) | Num 8 |  |  |  |
| ELIM_RHV | Elim reacto(HV) | Num 8 | 1 = subject eliminated from the ATP cohort for safety for elimination type |  |  |
| ELIM_RIE | Elim reacto(IE) | Num 8 | 1 = subject eliminated from the ATP cohort for safety for elimination type |  |  |
| ELIM_RIM | Elim reacto(IM) | Num 8 | 1 = subject eliminated from the ATP cohort for safety for elimination type |  |  |
| ELIM_RPT | Elim reacto(PT) | Num 8 | 1 = subject eliminated from the ATP cohort for safety for elimination type |  |  |
| ELIM_RRI | Elim reacto(RI) | Num 8 | 1 = subject eliminated from the ATP cohort for safety for elimination type |  |  |
| ELIM_RSM | Elim reacto(SM) | Num 8 | 1 = subject eliminated from the ATP cohort for safety for elimination type |  |  |
| ELIM_SCE | Elim sero(CE) | Num 8 | 1 = subject eliminated from the ATP cohort for immunogenicity for elimination type |  |  |
| ELIM_SCM | Elim sero(CM) | Num 8 | 1 = subject eliminated from the ATP cohort for immunogenicity for elimination type |  |  |
| ELIM_SHS | Elim sero(HS) | Num 8 | 1 = subject eliminated from the ATP cohort for immunogenicity for elimination type |  |  |
| ELIM_SHV | Elim sero(HV) | Num 8 | 1 = subject eliminated from the ATP cohort for immunogenicity for elimination type |  |  |
| ELIM_SIE | Elim sero(IE) | Num 8 | 1 = subject eliminated from the ATP cohort for immunogenicity for elimination type |  |  |
| ELIM_SIM | Elim sero(IM) | Num 8 | 1 = subject eliminated from the ATP cohort for immunogenicity for elimination type |  |  |
| ELIM_SPT | Elim sero(PT) | Num 8 | 1 = subject eliminated from the ATP cohort for immunogenicity for elimination type |  |  |
| ELIM_SRI | Elim sero(RI) | Num 8 | 1 = subject eliminated from the ATP cohort for immunogenicity for elimination type |  |  |
| ELIM_SSM | Elim sero(SM) | Num 8 | 1 = subject eliminated from the ATP cohort for immunogenicity for elimination type |  |  |
| ELIM_ST | Elim Stat | Num 8 |  |  |  |
| ELIM_V | Elim Vaccin | Num 8 |  |  |  |
| ELI_F3CE | Elim Efficacy(CE/3) | Num 8 | 1 = subject eliminated from the ATP population for efficacy for elimination type |  |  |
| ELI_F3CM | Elim Efficacy(CM/3) | Num 8 | 1 = subject eliminated from the ATP population for efficacy for elimination type |  |  |
| ELI_F3HS | Elim Efficacy(HS/3) | Num 8 |  |  |  |
| ELI_F3HV | Elim Efficacy(HV/3) | Num 8 | 1 = subject eliminated from the ATP population for efficacy for elimination type |  |  |
| ELI_F3IE | Elim Efficacy(IE/3) | Num 8 | 1 = subject eliminated from the ATP population for efficacy for elimination type |  |  |
| ELI_F3IM | Elim Efficacy(IM/3) | Num 8 | 1 = subject eliminated from the ATP population for efficacy for elimination type |  |  |
| ELI_F3PT | Elim Efficacy(PT/3) | Num 8 | 1 = subject eliminated from the ATP population for efficacy for elimination type |  |  |
| ELI_F3RI | Elim Efficacy(RI/3) | Num 8 | 1 = subject eliminated from the ATP population for efficacy for elimination type |  |  |
| ELI_F3SM | Elim Efficacy(SM/3) | Num 8 | 1 = subject eliminated from the ATP population for efficacy for elimination type |  |  |
| ELI_F4CE | Elim Efficacy(CE/4) | Num 8 | 1 = subject eliminated from the ITT population for elimination type |  |  |
| ELI_F4CM | Elim Efficacy(CM/4) | Num 8 |  |  |  |
| ELI_F4HS | Elim Efficacy(HS/4) | Num 8 | 1 = subject eliminated from the ITT population for elimination type |  |  |
| ELI_F4HV | Elim Efficacy(HV/4) | Num 8 |  |  |  |
| ELI_F4IE | Elim Efficacy(IE/4) | Num 8 | 1 = subject eliminated from the ITT population for elimination type |  |  |
| ELI_F4IM | Elim Efficacy(IM/4) | Num 8 |  |  |  |
| ELI_F4PT | Elim Efficacy(PT/4) | Num 8 |  |  |  |
| ELI_F4RI | Elim Efficacy(RI/4) | Num 8 |  |  |  |
| ELI_F4SM | Elim Efficacy(SM/4) | Num 8 |  |  |  |
| ELI_F5CE | Elim Efficacy(CE/5) | Num 8 | 1 = subject eliminated from the ATP population for efficacy for elimination type |  |  |
| ELI_F5CM | Elim Efficacy(CM/5) | Num 8 | 1 = subject eliminated from the ATP population for efficacy for elimination type |  |  |
| ELI_F5HS | Elim Efficacy(HS/5) | Num 8 | 1 = subject eliminated from the ATP population for efficacy for elimination type |  |  |
| ELI_F5HV | Elim Efficacy(HV/5) | Num 8 | 1 = subject eliminated from the ATP population for efficacy for elimination type |  |  |
| ELI_F5IE | Elim Efficacy(IE/5) | Num 8 | 1 = subject eliminated from the ATP population for efficacy for elimination type |  |  |
| ELI_F5IM | Elim Efficacy(IM/5) | Num 8 | 1 = subject eliminated from the ATP population for efficacy for elimination type |  |  |
| ELI_F5PT | Elim Efficacy(PT/5) | Num 8 | 1 = subject eliminated from the ATP population for efficacy for elimination type |  |  |
| ELI_F5RI | Elim Efficacy(RI/5) | Num 8 | 1 = subject eliminated from the ATP population for efficacy for elimination type |  |  |
| ELI_F5SM | Elim Efficacy(SM/5) | Num 8 | 1 = subject eliminated from the ATP population for efficacy for elimination type |  |  |
| F2200CAD | F2200CAD | Char 1 | N = No |  |  |
|  |  |  | Y = Yes |  |  |
| F2200OLD | F2200OLD | Char 1 | N = No |  |  |
|  |  |  | Y = Yes |  |  |
| F6000OLD | F6000OLD | Char 1 | N = No |  |  |
|  |  |  | Y = Yes |  |  |
| FATAL24_ | Fatal Malaria Secondary Case Def 4 | Num 8 |  |  |  |
| GROUP_NB | Group number | Num 8 | 1 = R3R |  |  |
|  |  |  | 2 = R3C |  |  |
|  |  |  | 3 = C3C |  |  |
| MORT1_ | All-cause mortality def 1 | Num 8 |  |  | See Analysis plan |
| MORT2_ | All-cause mortality def 2 | Num 8 |  |  |  |
| PID | Subject Identification | Num 8 |  |  | From *.pid field on the first page of the CRF |
| RND_ID | Treatment number | Num 8 |  |  | See pid.rnd_id |
| SCHEDULE | Schedule (clinical effective) | Num 8 |  |  | See pid.schedule |
| SITE | Site | Char 9 | Agogo |  | See pid.site |
|  |  |  | Bagamoyo |  |  |
|  |  |  | Kilifi |  |  |
|  |  |  | Kintampo |  |  |
|  |  |  | Kombewa |  |  |
|  |  |  | Korogwe |  |  |
|  |  |  | Lambarene |  |  |
|  |  |  | Lilongwe |  |  |
|  |  |  | Manhica |  |  |
|  |  |  | Nanoro |  |  |
|  |  |  | Siaya |  |  |
| TIMETOD | Time to death in months | Num 8 |  |  |  |
| TREATMNT | Treatment | Char 200 | Control |  | See pid.treatmnt |
|  |  |  | RTS,S/AS01E primary schedule with booster |  |  |
|  |  |  | RTS,S/AS01E primary schedule without booster |  |  |

Study 110021 - wunsol

| **Variable** | **Description** | **Type** | **Codes** | **Crf Page** | **Comment** |
| --- | --- | --- | --- | --- | --- |
| ACTIVITY | Activity | Num 8 | 231 = UNSOL2 |  | From *.activity field on top of first page of each CRF segment |
|  |  |  | 500 = SERIOUS AES |  |  |
|  |  |  | 51 = UNSOL1 |  |  |
| AELASTDO | Last dose administered | Num 8 |  |  | Derived: last study dose administered before or on the start of the AE. |
| AELASTVA | Previous vaccination | Num 8 |  |  | Derived: latest date when a study vaccine dose was administered BEFORE or on the start date of the AE. |
| AEMOD | Keyword (WHOAE) | Char 80 |  |  | From safety physician. When the verbatim of the adverse event (ae_desc) could not be matched to a lower level term of the WHO dictionary by the automatic encoder, a physician assigned a standardized description - matching entries in the WHO dictionary - manually. See (whoae). ALL VALUES MISSING |
| AEPOSTVC | AE during immediate post-vacc period | Char 2 | Y = Ae during immediate post-vaccination | crf1:71, 113 | ae.aepostvc |
| AE_AID | IMD / AID | Char 2 |  | crf1:163 | ae.ae_aid |
| AE_DAY_O | Onset Day | Num 8 |  |  | Derived: onset day of the AE relative to the last study dose administered (aelastdo). 0=on the day of study dose administration. |
| AE_DESC | AE Desciption | Char 80 |  | crf1:5, 71, 113, 163 | ae.ae_desc |
| AE_DUR | AE duration | Num 8 |  |  | Derived: duration of ae expressed in days and calculated as follows : (ae_erdat) - (ae_srdat) + 1 |
| AE_ERDAT | End date | Char 9 |  | crf1:5, 71, 113, 163 | ae.ae_erdat |
| AE_INTEN | Intensity | Num 3 | 1 = MILD | crf1:5, 71, 113, 163 | ae.ae_inten  As a numeric value. Missing confirmed (’MC’) have been coded as 9. |
|  |  |  | 2 = MODERATE |  |  |
|  |  |  | 3 = SEVERE |  |  |
|  |  |  | 9 = MISSING CONFIRMED |  |  |
| AE_LG | Local/General | Char 2 | G = NON-ADMINISTRATION SITE | crf1:71, 113 | ae.ae_lg |
|  |  |  | L = ADMINISTRATION SITE |  |  |
| AE_NB | AE No | Char 3 |  | crf1:71, 113 | ae.ae_nb |
| AE_SER | Serious Adverse ? | Char 2 | N = No |  | ae.ae_ser |
|  |  |  | Y = Yes |  |  |
| AE_SRDAT | Start date | Char 9 |  | crf1:5, 71, 113, 163 | ae.ae_srdat |
| AGEC | Age Group (N) | Num 8 | 1 = [5-17] months |  | See pid.agec |
|  |  |  | 2 = [6-12] weeks |  |  |
| BD_INTEN | Intensity database | Char 2 | 1 = MILD | crf1:5, 71, 113, 163 | ae.ae_inten |
|  |  |  | 2 = MODERATE |  |  |
|  |  |  | 3 = SEVERE |  |  |
|  |  |  | MC = MISSING CONFIRMED |  |  |
|  |  |  | NA = NOT APPLICABLE |  |  |
| BS_CODE | Body*syst | Num 8 |  |  | Derived: code of the body system class associated to the lower level code (whoae) according to the WHO dictionary. ALL VALUES MISSING |
| BS_DEC1 | Classification | Char 40 |  |  | Derived: label associated to the body system class code (bs_code) in the WHO dictionary. ALL VALUES MISSING |
| CAUSAL | Causality | Char 2 | N = Not related to study vaccine(s) | crf1:5, 71, 113, 163 | ae.causal |
|  |  |  | Y = Related to study vaccine(s) |  |  |
| CHRONIC | Chronic | Char 2 |  |  | NOT APPLICABLE |
| DB_CODE | Vaccine code blinded | Char 3 |  |  | Derived: for mono-vaccination activities, comes from vaccprod.p_code. For multi-vaccination activities, comes from ae.p_code. |
| ELIM_ITB | Elim (ITB) | Num 8 |  |  |  |
| ENDITT_BST | End date of FU period for cohort ITT | Num 8 |  |  |  |
| ENDITT_EE | End date of FU period for cohort ITT | Num 8 |  |  | End of ITT [M0-Dec13] |
| ENDITT_SE | End date of FU period for cohort ITT | Num 8 |  |  | End of ITT [M0-M32] |
| EXTENSION | EXTENSION | Num 8 |  |  | Consented to extension phase (M32-Dec13) |
| GROUP_NB | Group number | Num 8 | 1 = R3R |  | See pid.group_nb |
|  |  |  | 2 = R3C |  |  |
|  |  |  | 3 = C3C |  |  |
| HLGTCODE | Hlgt Code | Char 8 |  |  | Derived: higher level group code associated to the lower level code (md_code) according to the MedDRA dictionary. |
| HLGTNAME | hlgt name | Char 120 |  |  | Derived: label associated to the higher level group code (hlgtcode) in the MedDRA dictionary. |
| HLT_CODE | Hlt Code | Char 8 |  |  | Derived: higher level code associated to the lower level code (md_code) according to the MedDRA dictionary. |
| HLT_NAME | hlt name | Char 120 |  |  | Derived: label associated to the higher level code (hlt_code) in the MedDRA dictionary. |
| INTEN_L | Intensity decoded | Char 17 |  |  | Derived: decode of intensity |
| LLT_NAME | Lower Level Term name | Char 100 |  |  | Derived: label associated to the lower level code (llt_code) in the MedDRA dictionary. |
| MA_TYPE | Medical advice type | Char 2 | ER = EMERGENCY ROOM | crf1:5, 71, 113, 163 | ae.ma_type |
|  |  |  | HO = HOSPITALIZATION |  |  |
|  |  |  | MD = MEDICAL PERSONNEL |  |  |
| MD_CODE | Llt Code | Char 8 |  |  | Derived: code of the lower term of the MedDRA dictionary matching the description of the adverse event (ae_desc). Matching is performed by an automatic encoder. When the automatic encoder could not find a match, a physician standardized the description (ae_desc) into (md_mod). Then the automatic encoder assigned the code using (md_mod) for the match. |
| MD_MOD | Keyword (MedDRA) | Char 80 |  |  | From safety physician. When the verbatim of the adverse event (ae_desc) could not be matched to a lower level term of the MedDRA dictionary by the automatic encoder, a physician assigned a standardized description - matching entries in the MedDRA dictionary - manually. See (md_code). |
| MEDTY_L | Med Adv type decoded | Char 17 |  |  | Derived: decode of Med Adv type |
| MED_ADV | Did the subject seek medical advice? | Char 2 | MC = MISSING CONFIRMED | crf1:71, 113 | ae.med_adv  Y if medical advice |
|  |  |  | N = NO |  |  |
|  |  |  | Y = YES |  |  |
| MED_TYPE | Medically attended visit | Char 2 |  |  | Derived: NO if med_adv equal N, MD if med_adv equal Y and ma_type does not exist, equal ma_type if ma_type exists, missing in other cases |
| OUTCOME | Outcome | Char 2 | 1 = RECOVERED/RESOLVED | crf1:5, 71, 113, 163 | ae.outcome |
|  |  |  | 2 = RECOVERING/RESOLVING |  |  |
|  |  |  | 3 = NOT RECOVERED/NOT RESOLVED |  |  |
|  |  |  | 4 = RECOVERED/RESOLVED WITH SEQUELAE |  |  |
|  |  |  | 5 = FATAL |  |  |
|  |  |  | MC = MISSING CONFIRMED |  |  |
| OUTCO_L | Outcome decoded | Char 32 |  |  | Derived: decode of outcome |
| PID | Subject Identification | Num 8 |  |  | From *.pid field on the first page of the CRF |
| PIMD | pIMD | Char 1 |  |  | Derived: Y if AE classified as a pIMD based on MedDRA dictionary |
| PREF_COD | pt_whoae | Char 4 |  |  | Derived: code of the preferred term associated to the lower level code (whoae) according to the WHO dictionary. ALL VALUES MISSING |
| PREF_DEC | Preferred term | Char 70 |  |  | Derived: label associated to the preferred term code (pref_cod) in the WHO dictionary. ALL VALUES MISSING |
| PT_CODE | PT Code | Char 8 |  |  | Derived: code of the preferred term associated to the lower level code (md_code) according to the MedDRA dictionary. |
| PT_NAME | Preferred*term | Char 100 |  |  | Derived: label associated to the preferred term code (pt_code) in the MedDRA dictionary. |
| P_CODE | Vaccine code unblinded | Char 3 |  |  | Derived: internal vaccine code after unblinding. Unblinded value is based on the blinded code (vaccprod.p_code) and the treatment group (pid.group_nb). |
| RELVACDO | Activity related vaccine dose | Num 8 | 1 |  | From protocol. Vaccine dose planned at the activity in the protocol. So this dose number is directly related to the activity irrespective of whether the dose was administered or not. |
|  |  |  | 2 |  |  |
|  |  |  | 3 |  |  |
|  |  |  | 4 |  |  |
| SAE_NB | SAE No | Char 4 |  | crf1:5, 163 | ae.sae_nb |
| SCHEDULE | Schedule (clinical effective) | Num 8 |  |  | See pid.schedule |
| SITE | Site | Char 9 | Agogo |  | See pid.site |
|  |  |  | Bagamoyo |  |  |
|  |  |  | Kilifi |  |  |
|  |  |  | Kintampo |  |  |
|  |  |  | Kombewa |  |  |
|  |  |  | Korogwe |  |  |
|  |  |  | Lambarene |  |  |
|  |  |  | Lilongwe |  |  |
|  |  |  | Manhica |  |  |
|  |  |  | Nanoro |  |  |
|  |  |  | Siaya |  |  |
| SOC_CODE | SOC Code | Char 8 |  |  | Derived: code of the System Organ Class associated to the lower level code (md_code) according to the MedDRA dictionary. |
| SOC_NAME | SYSTEM ORGAN CLASS | Char 120 |  |  | Derived: label associated to the System Organ Class code (soc_code) in the MedDRA dictionary. |
| START | Start date | Num 8 |  |  | Derived: ae.ae_srdat in SAS date format |
| STRTITB | Start date of FU period for cohort ITB | Num 8 |  |  | Start of follow up ITT [M20-Dec13] |
| STRTITT | Start date of FU period for cohort ITT | Num 8 |  |  | Start of follow up ITT [M0-Dec13] |
| STR_DATE | STR_DATE | Num 8 |  |  |  |
| WHOAE | Who*Code | Char 8 |  |  | Derived: code of the lower term of the WHO dictionary matching the description of the adverse event (ae_desc). Matching is performed by an automatic encoder. When the automatic encoder could not find a match, a physician standardized the description (ae_desc) into (aemod). Then the automatic encoder assigned the code using (aemod) for the match. ALL VALUES MISSING |
